# Fractal Characterization of Simulated Metal Nanocatalysts in 3D

**DOI:** 10.1002/smsc.202400123

**Published:** 2024-07-09

**Authors:** Jonathan Y. C. Ting, George Opletal, Amanda S. Barnard

**Affiliations:** ^1^ School of Computing Australian National University 145 Science Road Canberra ACT 2601 Australia; ^2^ Data61 Commonwealth Scientific and Industrial Research Organisation Melbourne VIC 3008 Australia

**Keywords:** fractal dimension, metal nanoparticles, structural features, surface roughness

## Abstract

The surface roughness of metal nanoparticles is known to be influential toward their properties, but the quantification of surface roughness is challenging. Given the recent availability of large‐scale simulated data and tools for the computation of the box‐counting dimension of simulated atomistic objects, researchers are now enabled to study the connections between the surface roughness of metal nanoparticles and their properties. Herein, the relationships between the fractal box‐counting dimension of metal nanoparticle surfaces and structural features relevant to experimental and computational studies are investigated, providing actionable insights for the manufacturing of rough nanoparticles. This approach differs from conventional concepts of roughness, but introduces a possible indicator for their functionalities such as catalytic performance that was not previously accessible. It is found that, while it remains difficult to consistently correlate the dimension with the catalytic activity of surface facets, matching trends with their surface energy, thermodynamic stability, and number of bond vacancy are observed. This highlights the potential of fractal box‐counting dimensions to rationalize catalytic activity trends among metal nanoparticles, and opens up opportunities for the design of nanocatalysts with better performance via surface engineering.

## Graphical abstract

The surface roughness of metallic nanoparticles is an indicator of their catalytic activity. Herein, a general approach is presented using the fractal dimension, which can be applied to 2D and 3D data. This measure is also correlated with a number of well‐established properties that can be extracted from conventional computational modeling.

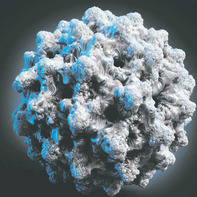

## 1 Introduction

Metal nanoparticles are known as ideal catalysts for various industrially important reactions,^[^ ^1^, ^2^ ^]^ and it is well known that the catalytic activities of metal nanoparticles are impacted by their surface roughness.^[^ ^3^, ^4^ ^]^ Rougher surfaces have greater surface area and can favor adsorption of certain chemical species, such as the hydroxyls on rough copper–platinum surfaces^[^ ^5^ ^]^ and the O(H)* intermediates on platinum surfaces^[^ ^6^ ^]^ for oxygen reduction reactions.

Despite the recognized importance of surface roughness, it has been difficult to measure it quantitatively for metal nanoparticles. This is partly due to the limitations of the experimental equipment in measuring the surface properties at high resolution. Additionally, metal nanoparticles are often not in equilibrium with their environment,^[^ ^7^ ^]^ causing properties such as their surface structures to vary based on the environmental conditions.^[^ ^3^, ^7^ ^]^ In electrochemical reactions, metal nanoparticles may undergo leaching of active metals, surface geometric restructuring, and surface compositional restructuring,^[^ ^3^, ^8^ ^]^ all of which alter the roughness. The frequent temporal changes in the surface structures of metal nanoparticles exacerbate the difficulty in capturing the information about these important structure/property relationships.

With the development of efficient computational methods and wide availability of computational resources, researchers are now enabled to conduct studies on metal nanoparticles in silico. In computational studies, these nanoparticles are often modeled as collections of spheres sized according to their atomic or molecular radii.^[^ ^9^, ^10^ ^]^ The resulting surfaces of these objects are depicted as complex shapes with intricate details due to overlapping spheres, including protrusions (or convexities) and indentations (or concavities) on the surface,^[^ ^11^ ^]^ and contribute to the overall surface roughness. This determines the amount of surface area available for interaction with other entities, which are the reactants in the case of the applications of metal nanoparticles as catalysts.^[^ ^12^, ^13^ ^]^

Previous studies focused on generating overly simplified or idealized hypothetical surfaces of metal nanoparticles to demonstrate certain generalizable trends among the nanoparticles of interest. Rough nanoparticles are commonly modeled either as regularly shaped grooves^[^ ^14^, ^15^, ^16^ ^]^ or by simply adding stochastic noises to the investigated properties of a smooth surface.^[^ ^17^, ^18^ ^]^ However, these approaches do not provide a deeper understanding on how the surface roughness of metal nanoparticles relates to their properties and suffers from a lack of realism. A quantitative measure that can capture the details of the protrusions and indentations of individual (physically realistic) metal nanoparticle surfaces accurately and effectively could assist in the design of more efficient metal nanoparticle catalysts.

Given the coordinates of the individual atoms in an atomistic object, the entire surface can be defined by assuming that each atom is a sphere that overlaps with neighboring atom spheres. This collective surface can be treated as a 3D object, which can then be characterized by the surface roughness parameters from the shape complexity research domain. One such parameter is fractal dimension,^[^ ^19^ ^]^ which is a dimensionless, unitless real‐value index that quantifies the complexity of an object as ratio of change in detail to change in scale. Fractal dimension can be defined and be measured empirically in connection with real‐world data, and has been used to characterize galaxies,^[^ ^20^ ^]^ landscapes,^[^ ^21^ ^]^ coastlines,^[^ ^22^ ^]^ clouds,^[^ ^23^ ^]^ lightnings,^[^ ^24^ ^]^ cerebella,^[^ ^25^ ^]^ cauliflowers,^[^ ^26^ ^]^ and leaves.^[^ ^27^ ^]^

A common method to estimate the fractal dimension of an object is by computing its box‐counting dimension ($D_{B}$)^[^ ^28^ ^]^ In this approach, the space containing the object is broken into increasingly smaller boxes with side length *ε*, and the number of boxes containing patterns of interest N(ε) is counted at each scale. The $D_{B}$ value is then computed as the slope of the log–log plot of change in box counts with respect to the scaling of the box lengths (Equation (1)).

(1) $$D_{B}={\text{lim}}_{\varepsilon \to 0}\frac{\text{log}(N(\varepsilon ))}{\text{log}(1/\varepsilon )}$$

The calculation of the $D_{B}$ of the surfaces of individual metal nanoparticles has been made possible by a recently developed tool adapted to simulated atomistic objects.^[^ ^29^ ^]^ The capability of the tool in capturing the surface roughness of given atomic coordinates quantitatively was demonstrated on a limited set of palladium nanoparticles. The questions then remain as to how this approach scales to larger and more complex metal nanocatalysts, and whether the dimensionality is related to characteristics that can be accessed in the labs.

In previous works, the box‐counting dimensions of titania and ceria nanoparticles computed from their transmission electron miscroscopy (TEM) images provided information about the growth mechanism of hard‐aggregates and the surface chemistry of agglomerated nanoparticles, which aided in the quality control of the synthesis process.^[^ ^30^ ^]^ Ganguly, Basu, and Sikdar also computed the fractal dimensions of aggregated aluminum oxide nanoparticles in colloidal nanofluids from their scanning electron microscopy (SEM) images by analyzing the relationships between length of squares and area occupied by aggregated clusters to obtain quantitative information about their morphological features.^[^ ^31^ ^]^ However, these approaches applied were limited to 2D images of nanoparticle aggregates. This work differs in that we aim to estimate the fractal dimension of 3D surfaces of individual metal nanoparticles, which provides much more informative insights regarding their roughness. The roughness portrayed in 2D images could potentially provide an incomplete picture of the actual 3D surfaces and be misleading. The novelty of this work hence lies in the connection made between the 3D fractal dimension of the surfaces and their structural features.

In this study, we compute the $D_{B}$ of a set of simulated monometallic, bimetallic, and trimetallic nanoparticles consisting of combinations of gold (Au), palladium (Pd), and platinum (Pt), and study their relationships with structural features including nanoparticle size, fraction of atoms on specific surface facets, surface curvature, and crystallinity. Actionable insights are obtained based on the observation of the characteristics of rough nanoparticles. We subsequently explored the connection between $D_{B}$ and the catalytic activities of different surface facets of metal nanoparticles, and discovered that while it is difficult to directly relate $D_{B}$ to catalytic activities in a consistent manner, $D_{B}$ holds the potential to be a cheaper alternative to the computationally expensive (but essential) indicators currently used in for catalysis studies, such as surface energy, thermodynamic stability, or *d*‐band theory‐based methods.^[^ ^32^ ^]^

## 2 Results and Discussion

### 2.1 Data Preparation

The nanoparticle datasets used here were not produced as part of this study, but are publicly available.^[^ ^33^, ^34^, ^35^, ^36^, ^37^, ^38^, ^39^, ^40^, ^41^, ^42^ ^]^ Readers are directed to Section 4.1 for further details about the raw datasets, the contents of which are summarized in Table 2.

Prior to the analysis, the individual Au, Pd, and Pt datasets are combined into a single monometallic nanoparticle (MNP) dataset, with new columns designating the element‐specific features. For example, AuAu, PdPd, and PtPt bond length statistics are added to the MNP dataset, in addition to the original (symbolized as MM) bond length statistics.

Due to the much larger number of samples of the bimetallic nanoparticle (BNP) datasets, the redundant nanoparticles (data instances) in the original datasets are first identified and removed prior to any merging. The assessment of redundancy is based on a set of similarity metrics, with thresholds determined based on observations on a chosen subset of nanoparticles. Further details regarding the metrics and thresholds are included in the Supporting Information. Among the six BNP datasets, three of them (AuPd, AuPt, and PdPt) are composed of similar elemental combinations as the other three (PdAu, PtAu, and PtPd), but contain different patterning of the elements (e.g., AuPd contains Au@Pd core–shell nanoparticles but Pd@Au do not). Therefore, the BNP datasets consisting of the same elements were merged prior to the combination of the individual datasets (AuPd, AuPt, and PdPt) into a single BNP dataset using the same procedure as the MNP datasets. In addition to this, a single trimetallic nanoparticle (TNP) dataset is used, containing different combinations of AuPdPt.

**Figure 1:**
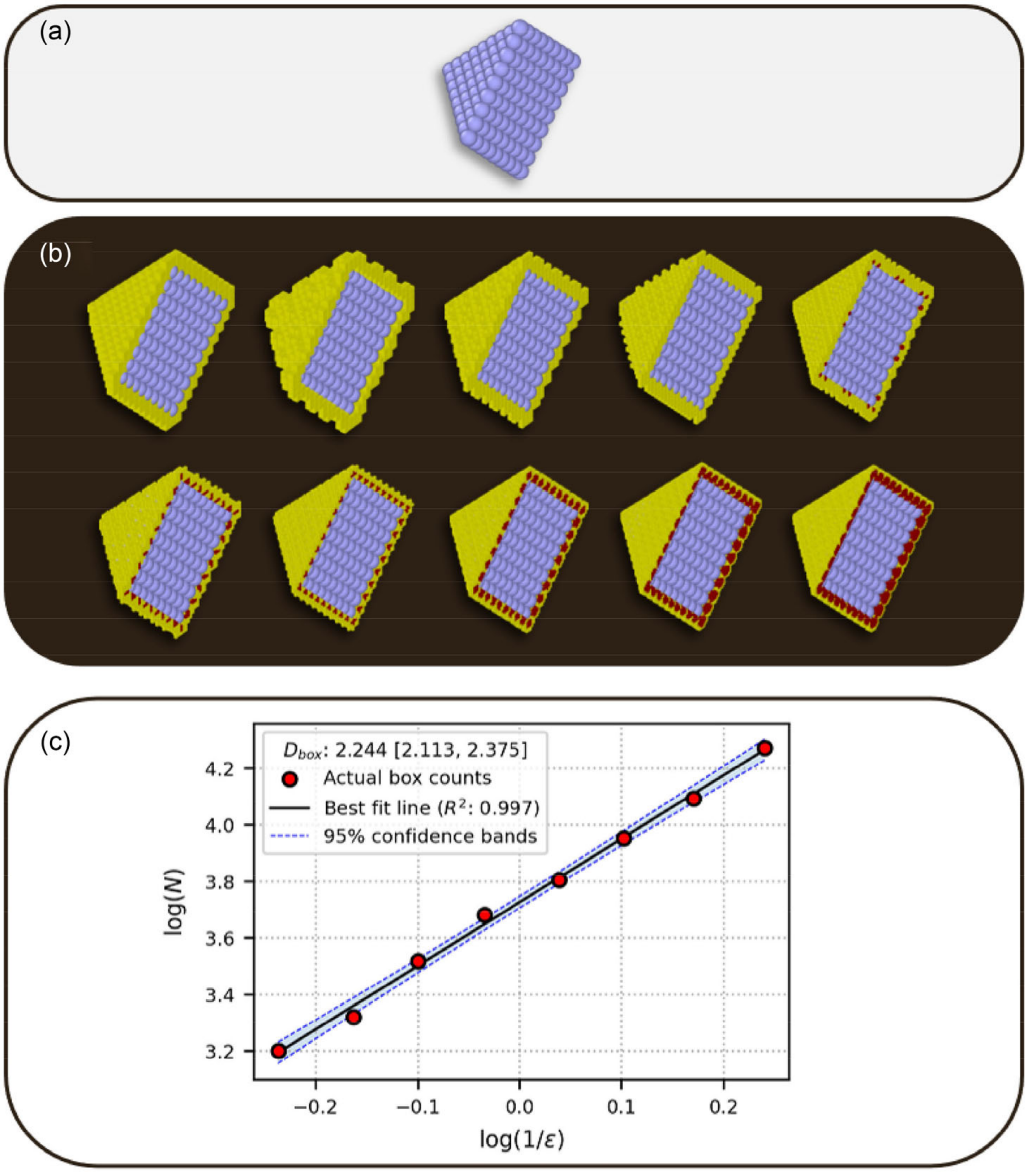
Results from applying the box‐counting algorithm on a Pd nanoparticle, turning a) a mathematically exact representation of its surface into b) box‐counts at different scales, and c) fitting a linear regression to the results.

The $D_{B}$ values of the metal nanoparticles are computed using the methodology outlined under Section 4.2. **Figure** **1** illustrates the process involved in the computation of $D_{B}$ on an exemplar nanoparticle.

At this point it is important to note that the roughness quantified by $D_{B}$ is not necessarily the same as that commonly understood by the catalysis community, where roughness is being regarded as the deviation of surface heights from an average value.^[^ ^17^, ^43^, ^44^, ^45^ ^]^ The computation of $D_{B}$ is known to be sensitive to certain parameters, including the range and interval of the box sizes, and the orientation of the boxes with respect to the objects.^[^ ^46^, ^47^ ^]^ The particular choices of parameters used to compute the $D_{B}$ for this work are determined based on experiments on a set of nanoparticles, as detailed under Section 4.2. In this case, $D_{B}$ is closer to the concept in the work of Hsieh et al. where roughness is defined as the ratio of surface area to a reference smooth surface.^[^ ^48^ ^]^

Following this section, we analyze the distributions of $D_{B}$ across nanoparticles with different features in Section 2.2. The relationships between $D_{B}$ and the structural features are investigated in Section 2.3. Finally, we report our findings on the comparison of our observations with the literature in Section 2.4.

### 2.2 Distribution of Box‐Counting Dimensions

#### 2.2.1 Elemental Combination

**Figure 2:**
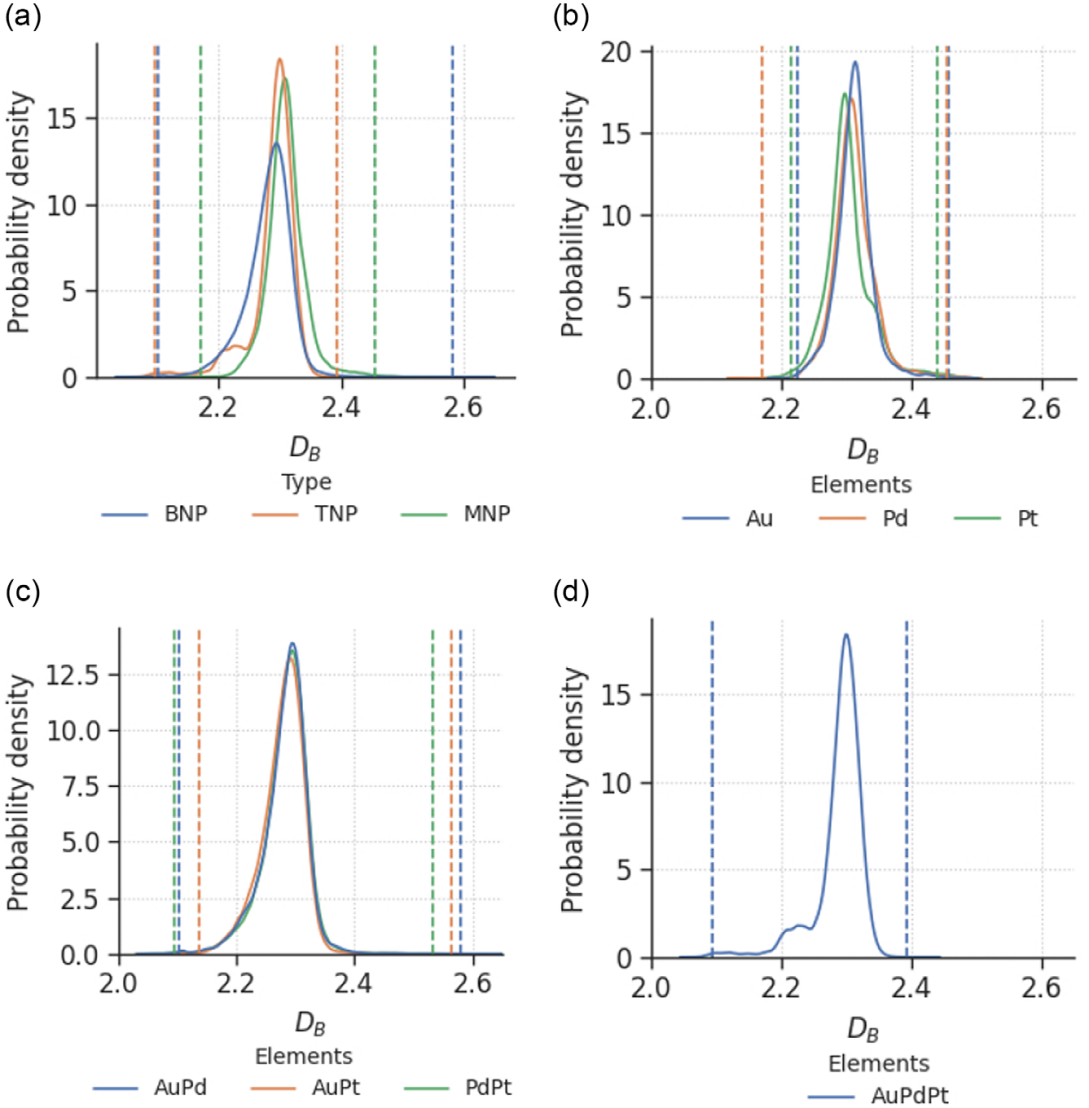
Probability density distributions of box‐counting dimensions for a) all, b) monometallic, c) bimetallic, and d) trimetallic nanoparticles, where the areas under the curves sum up to 1. The vertical lines indicate the top and bottom 10% threshold for defining smooth and rough nanoparticles.

**Figure** **2** shows the distributions of $D_{B}$ for all monometallic, bimetallic, and trimetallic nanoparticles. Their distributions look similar, especially for all three types of elemental combinations of BNPs, which overlap almost perfectly with each other, having a Gaussian shape with a peak at 2.29. This is largely reflective of the data‐generation process, as the BNP nanoparticles were generated, sampled, and reduced using the same pipeline. Readers are directed to Section 4.1 for further details and references.

**Figure 3:**
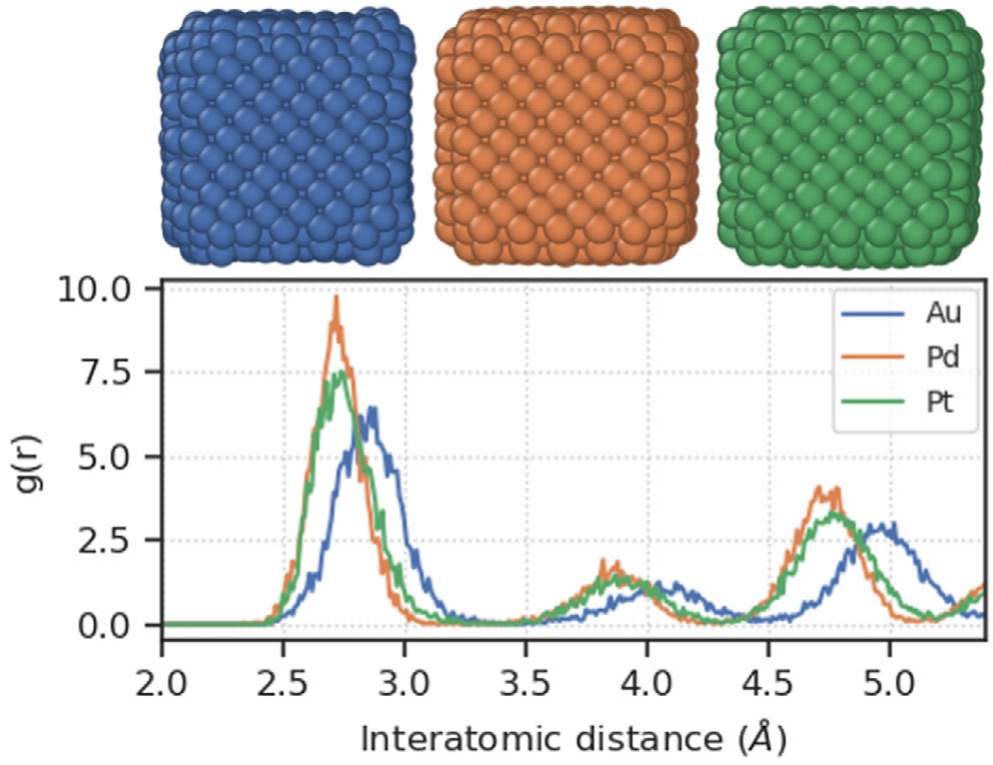
Surface structures (upper) and radial distribution functions (lower) of cubic Au (left), Pd (middle), and Pt (right) nanoparticles. The gaps between atoms decrease following the trend of Au > Pd > Pt.

For MNPs, the peaks of the distributions are slightly different, with Au nanoparticles having rougher surfaces (higher $D_{B}$) than Pd nanoparticles on average, which have rougher surfaces than Pt nanoparticles. The visualization of cubic nanoparticles consisting of the three elements (sampled from the lowest simulation temperature in the respective datasets) in **Figure** **3** revealed that the trend observed can be attributed to the differences in the surface structures due to the atomic radii (1.74 Å for Au, 1.69 Å for Pd, and 1.77 Å for Pt) and average bond lengths (2.8 Å for Au and 2.7 Å for Pd and Pt). These structures correspond to larger gaps between the atoms in Au nanoparticles compared to Pd and Pt nanoparticles, which, in turn, result in lower overall amount of grooves (concavities and convexities) that are captured by the box‐counting algorithm.

Upon comparison with the most relevant literature, we observe an opposite trend from the work of Melo, where Pt thin films were found to be rougher than Au films of similar thickness (40–100 nm).^[^ ^49^ ^]^ A possible cause for this discrepancy is the differences in the surface structures of nanoparticles (0D^[^ ^50^ ^]^) and nanofilms (2D^[^ ^51^, ^52^ ^]^), as suggested by Bannenberg et al. based on their comparison of these systems on their hydrogen sensing capabilities.^[^ ^53^ ^]^

Figure 2a shows that alloying has an effect of smoothing the surface roughness of the nanoparticles. While it is well understood that the introduction of lattice mismatches into nanoparticle structures results in surface vacancies and disordered areas,^[^ ^54^ ^]^ our results indicate that such disorder actually lowers the dimensionality (amount of space occupied by the outer surfaces as defined by the spherical atom assumption) of the nanoparticle. It is also noteworthy that a large proportion of the TNPs have surfaces that are rougher than the BNPs. This is attributed to size effect (nanoparticles within a particular size range having rougher surfaces) because the nanoparticles in our trimetallic dataset have a narrower size range compared to the BNPs, as described under Section 4.1.

#### 2.2.2 Alloying Ratio

**Figure 4:**
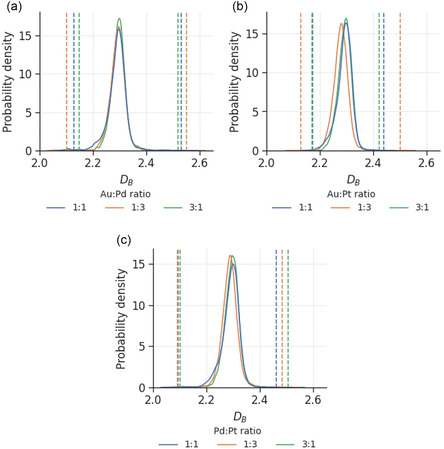
Probability density distribution of box‐counting dimensions for a) AuPd, b) AuPt, and c) PdPt with different alloying ratios, where the areas under the curves sum up to 1. The vertical lines indicate the top and bottom 10% threshold for defining smooth and rough nanoparticles.

In this section, we investigate the effect of modifying the alloying ratio on the nanoparticle $D_{B}$. **Figure** **4** shows the distributions of $D_{B}$ for BNPs with three different alloying ratios. Given the same processing conditions, the $D_{B}$ distributions look similar for all elemental combinations, indicating that $D_{B}$ does not depend significantly on the relative amount of each element. This opens up the possibility of reducing the costs required for the manufacturing of multimetallic nanocatalysts, by replacing more expensive elements with cheaper alternatives without compromising the complexity or roughness of their surfaces. Nevertheless, considering that catalytic processes are primarily facilitated by surface atoms, it is prudent to consider the impact of surface segregation on $D_{B}$.

#### 2.2.3 Surface Segregation

**Figure 5:**
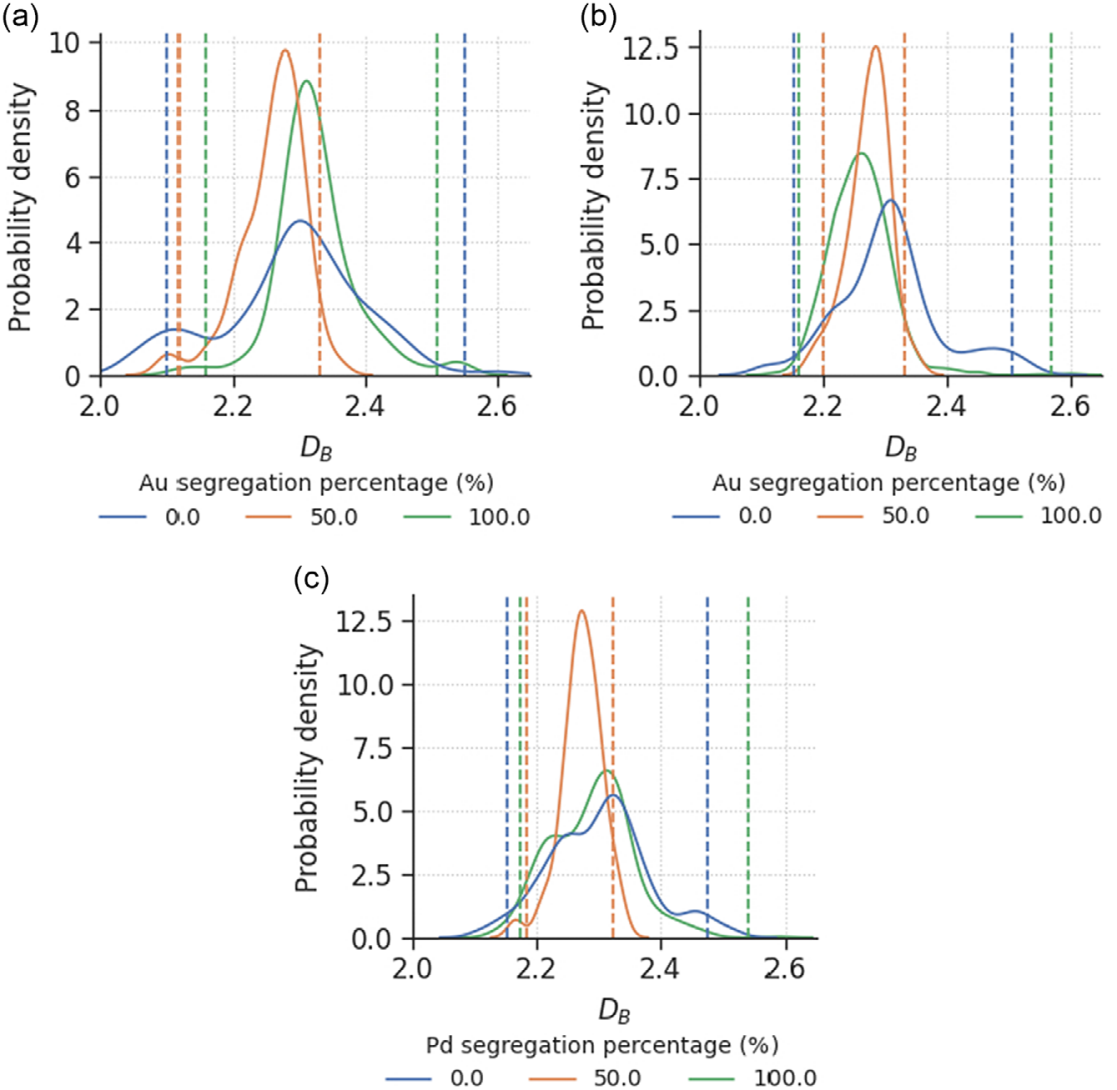
Probability density distribution of box‐counting dimensions for a) AuPd, b) AuPt, and c) PdPt nanoparticles with different degrees of surface segregation, where the areas under the curves sum up to 1. The vertical lines indicate the top and bottom 10% threshold for defining smooth and rough nanoparticles.

**Figure 6:**
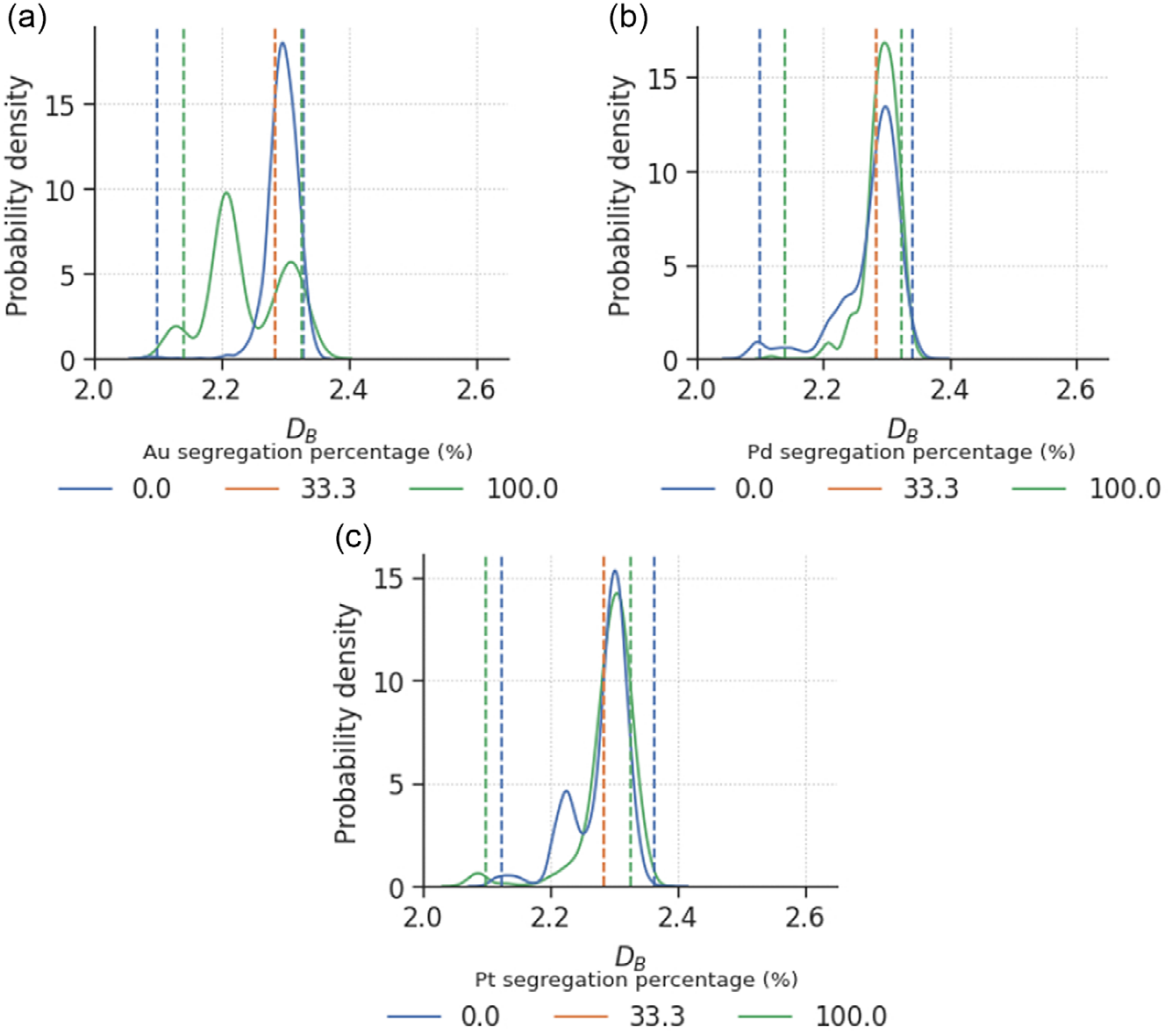
Probability density distribution of box‐counting dimensions for AuPdPt nanoparticles with different degrees of a) Au, b) Pd, and c) Pt surface segregation, where the areas under the curves sum up to 1. The vertical lines indicate the top and bottom 10% threshold for defining smooth and rough nanoparticles. There is only one nanoparticle that satisfies the 1:1:1 elemental ratio on the surface.

A comparison of the distributions of $D_{B}$ for BNPs with different degrees of surface segregation is shown in **Figure** **5**. Here, we can see that the nanoparticles with balanced 1:1 elemental ratio on the surface exhibit narrower distributions than the nanoparticles with surfaces completely covered by the same element. The roughest (top 10%) nanoparticles with complete surface segregation tend to be significantly rougher compared to nanoparticles without segregation, by at least 32% of the range of $D_{B}$ covered. The same effect was seen for TNPs in **Figure** **6**, where the completely segregated nanoparticles are found to be rougher than the nanoparticle with 1:1:1 elemental ratio of the surface. This is consistent with the observation made from the alloying effect in Section 2.2.1, where disorder on the surface was found to have a smoothening effect. Based on this finding, we can infer that rougher nanoparticles tend to be produced via synthesis methods and processing conditions known to induce further surface segregation, which typically include treatment with high temperature and allowing of the formation of larger nanoparticles.^[^ ^55^ ^]^

#### 2.2.4 Temperature

**Figure 7:**
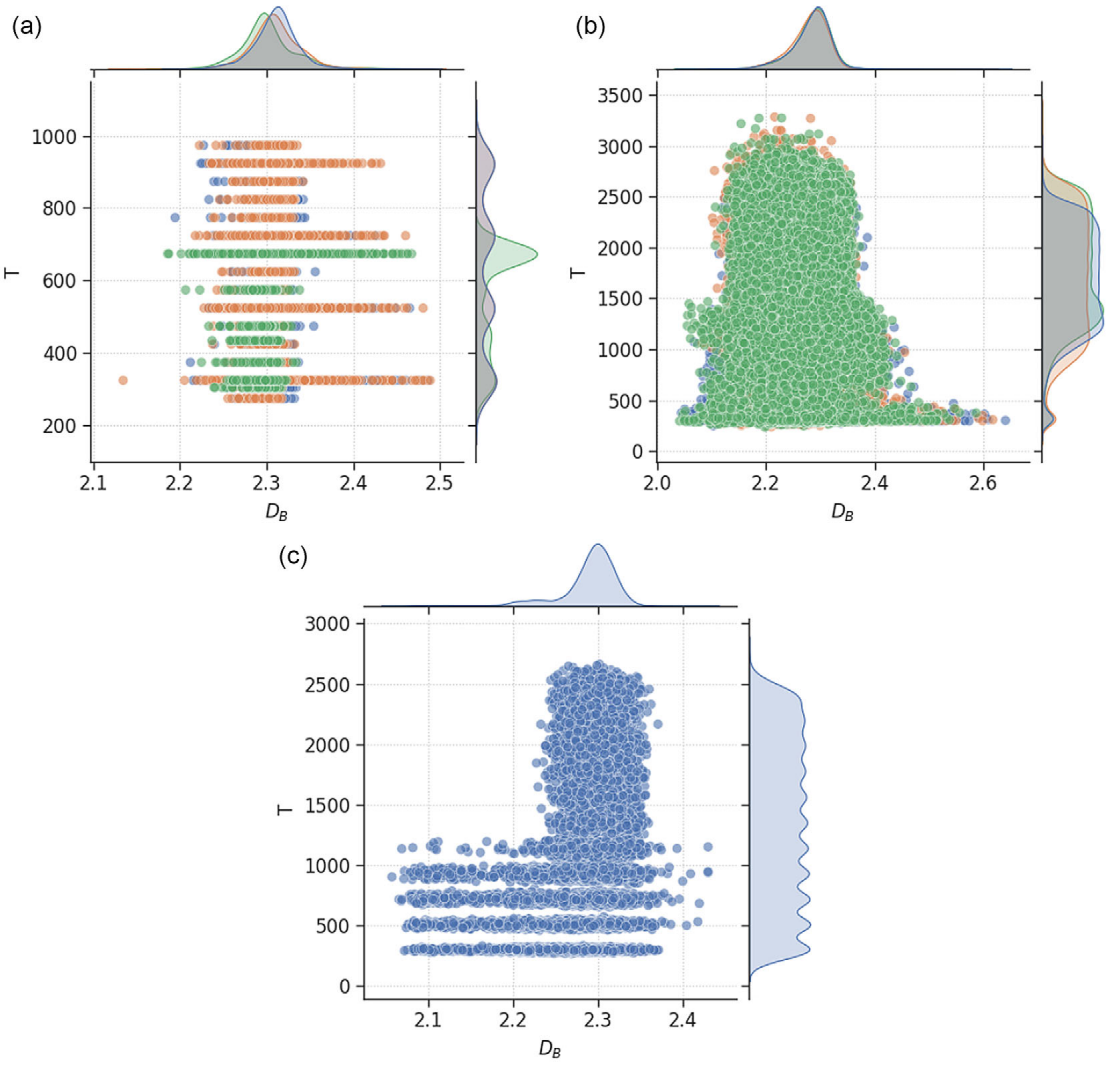
Scatter plot of box‐counting dimension and temperature for a) monometallic (with Au shown in blue, Pd in orange and Pt in green), b) bimetallic (with AuPd shown in blue, AuPt in orange and PdPt in green), and c) trimetallic (blue) nanoparticles. The mutual information scores for the relationships are provided in Table 1, with higher values indicating greater dependencies of the feature on box‐counting dimension.

The relationship between $D_{B}$ with temperature is one of the most crucial factors in the synthesis of metal nanoparticles. **Figure** **7** shows the relationship between $D_{B}$ of nanoparticles and the simulation temperatures at which the nanoparticles are sampled from. For MNPs, the scatter plots for Au and Pd nanoparticles show quadmodal distributions with equally tall peaks, reflecting the similar methodology used to generate the data, whereas Pt nanoparticles distributions have three peaks with a dominant peak at 623 K. The roughest BNPs are mostly sampled from relatively low simulation temperatures (<400, <500, and <1200 K for MNPs, BNPs, and TNPs, respectively). This indicates that increasing disorder in the crystal structure due to higher temperature tends to smooth out the nanoparticle surfaces, which is intuitive and consistent with domain knowledge.

### 2.3 Relating Dimensionality With Nanoparticle Structure

In the following sections, we investigate the relationship between $D_{B}$ with experimentally measurable metal nanoparticle structural features, typically accessible via techniques such as scanning transmission electron microscopy, electron energy‐loss spectroscopy, and energy dispersive X‐ray spectroscopy, and also computationally relevant features that are more difficult to measure experimentally. The mutual information between the features and $D_{B}$ are computed to capture the potentially nonlinear dependencies of the features on $D_{B}$. **Table** **1** lists the mutual information between the features investigated and $D_{B}$. The definitions of the features are listed in the Supporting Information. Further details related to the calculation of mutual information scores and assessment of their significance can be found under Section 4.5.

**Table 1:** Mutual information scores between the features investigated and box‐counting dimensions of the nanoparticles. The highest scores are highlighted in bold. The statistically insignificant relationships are highlighted in italics.

Feature	Dataset									Figure
	MNP				BNP				TNP	
	All	Au	Pd	Pt	All	AuPd	AuPt	PdPt	AuPdPt	
T	0.12	0.10	0.14	0.22	0.04	0.07	0.03	0.06	**0.41**	7
D_avg	0.15	0.20	0.17	0.27	0.40	**0.44**	0.41	0.41	0.38	8
Frac_FCC	0.11	0.13	0.13	0.16	0.23	0.25	0.19	0.23	**0.49**	9, S2, S3, S4
Frac_HCP	0.08	0.07	0.11	0.23	0.26	0.25	0.21	0.23	**0.36**	9, S2, S3, S4
Frac_ICOS	0.02	0.02	0.02	*0.00*	**0.31**	**0.31**	0.25	0.29	0.30	9, S2, S3, S4
Frac_DECA	0.09	0.08	0.12	0.24	0.29	0.29	0.25	0.26	**0.31**	9, S2, S3, S4
q6q6_S_avg	0.14	0.15	0.16	0.27	0.12	0.15	0.12	0.13	**0.51**	11
q6q6_B_avg	0.15	0.15	0.16	0.30	0.12	0.14	0.10	0.13	**0.50**	S5
q6q6_T_avg	0.11	0.12	0.12	0.20	0.13	0.16	0.12	0.13	**0.49**	S5
Frac_S_100	0.24	0.25	0.30	**0.33**	0.07	0.08	0.05	0.09	0.16	14, S6, S7, S8
Frac_S_110	0.14	0.12	0.18	0.22	0.15	0.16	0.14	0.15	**0.41**	14, S6, S7, S8
Frac_S_111	0.18	0.14	0.21	0.31	0.14	0.16	0.16	0.16	**0.41**	14, S6, S7, S8
Frac_Curve_1‐10	0.28	0.24	0.36	**0.38**	0.15	0.17	0.17	0.14	0.37	15, S9, S10, S11
Frac_Curve_11‐20	0.14	0.15	0.11	0.27	0.08	0.11	0.07	0.08	**0.47**	15, S9, S10, S11
Frac_Curve_21‐30	0.20	0.15	0.24	0.33	0.07	0.10	0.04	0.06	**0.39**	15, S9, S10, S11
Frac_Curve_31‐40	0.27	0.24	0.29	0.34	0.12	0.16	0.14	0.10	**0.45**	15, S9, S10, S11
Frac_Curve_41‐50	0.17	0.14	0.20	0.26	0.12	0.16	0.12	0.12	**0.34**	15, S9, S10, S11
Frac_Curve_51‐60	0.14	0.12	0.16	0.21	0.17	0.20	0.16	0.17	**0.31**	15, S9, S10, S11
Frac_Curve_61‐70	0.09	0.09	0.09	**0.26**	0.12	0.14	0.11	0.13	0.19	15, S9, S10, S11
Frac_Curve_71‐80	**0.02**	**0.02**	0.01	*0.00*	0.01	0.01	0.01	0.01	0.01	15, S9, S10, S11
MM_BL_avg	0.11	0.12	0.10	0.24	0.12	0.15	0.18	0.26	**0.35**	S12
MM_BL_std	0.11	0.16	0.14	0.28	0.09	0.13	0.07	0.17	**0.40**	S12
MM_BA1_avg	0.13	0.22	0.10	0.19	0.38	**0.43**	0.38	0.39	0.35	S13
MM_BA1_std	0.11	0.14	0.12	0.13	0.27	0.32	0.24	0.30	**0.34**	S13
MM_SCN_avg	0.11	0.12	0.14	0.23	0.21	0.28	0.19	0.25	**0.49**	S14
MM_BCN_avg	0.11	0.12	0.08	0.37	0.15	0.17	0.18	0.17	**0.48**	S14
MM_TCN_avg	0.17	0.19	0.14	0.17	0.32	0.38	0.30	0.34	**0.46**	S14
N_atom_total	0.16	0.22	0.19	0.28	0.39	**0.43**	0.37	0.39	0.15	S15
Volume	*0.00*	*0.00*	*0.00*	*0.00*	**0.41**	0.43	0.41	0.40	0.24	S16

#### 2.3.1 Nanoparticle Structure

**Figure 8:**
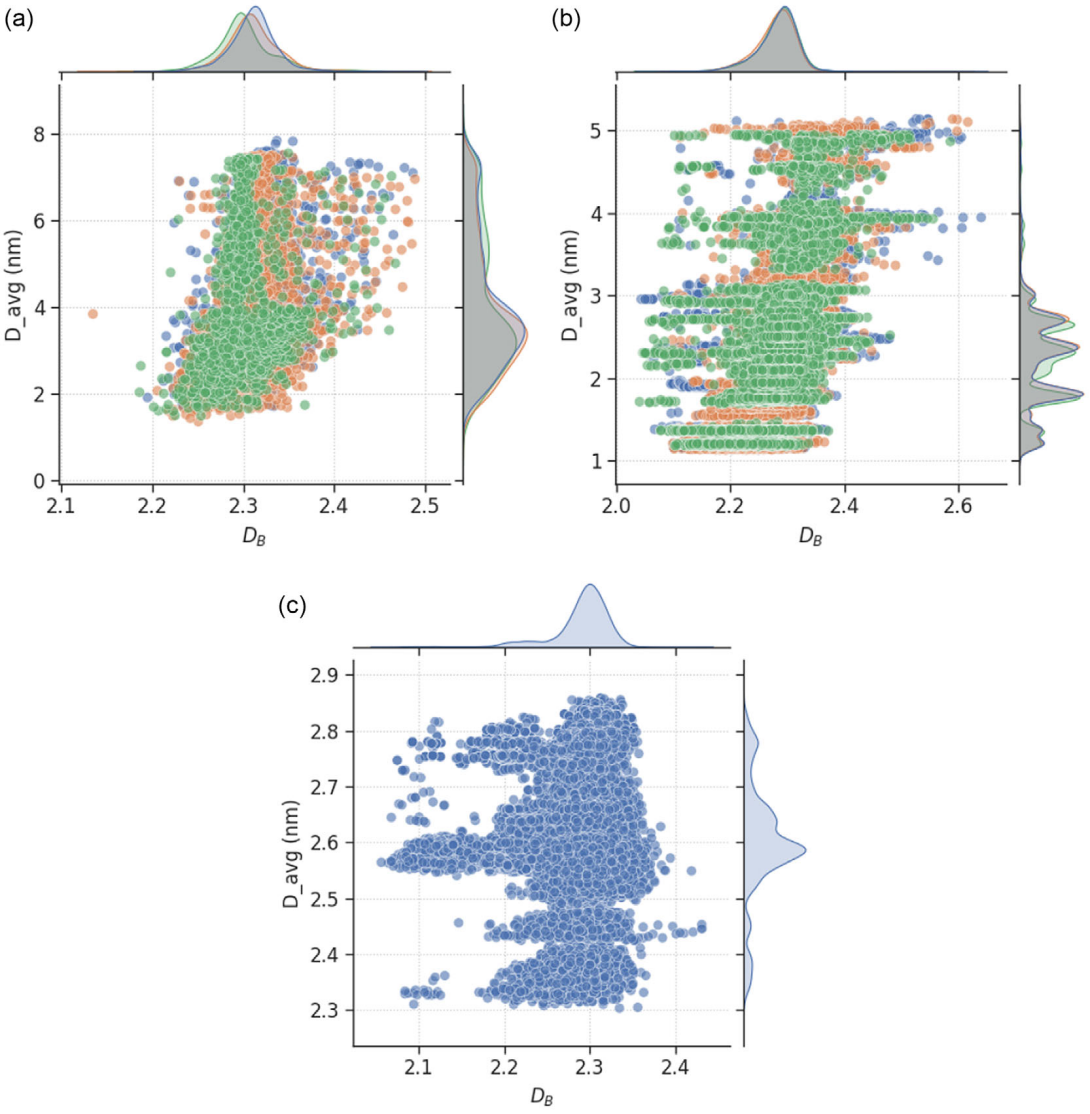
Scatter plot of box‐counting dimension and average diameter for a) monometallic (with Au shown in blue, Pd in orange and Pt in green), b) bimetallic (with AuPd shown in blue, AuPt in orange and PdPt in green), and c) trimetallic (blue) nanoparticles. The mutual information scores for the relationships are provided in Table 1, with higher values indicating greater dependencies of the feature on box‐counting dimension.

One of the most well studied factors in the synthesis of metal nanoparticles is the size. We show in **Figure** **8** the relationship between $D_{B}$ of nanoparticles and their average diameters. The plots indicate that rougher nanoparticles tend to be larger in size, in agreement with the report in the literature that surface segregation is promoted by increasing the size of nanoparticles.^[^ ^56^ ^]^

**Figure 9:**
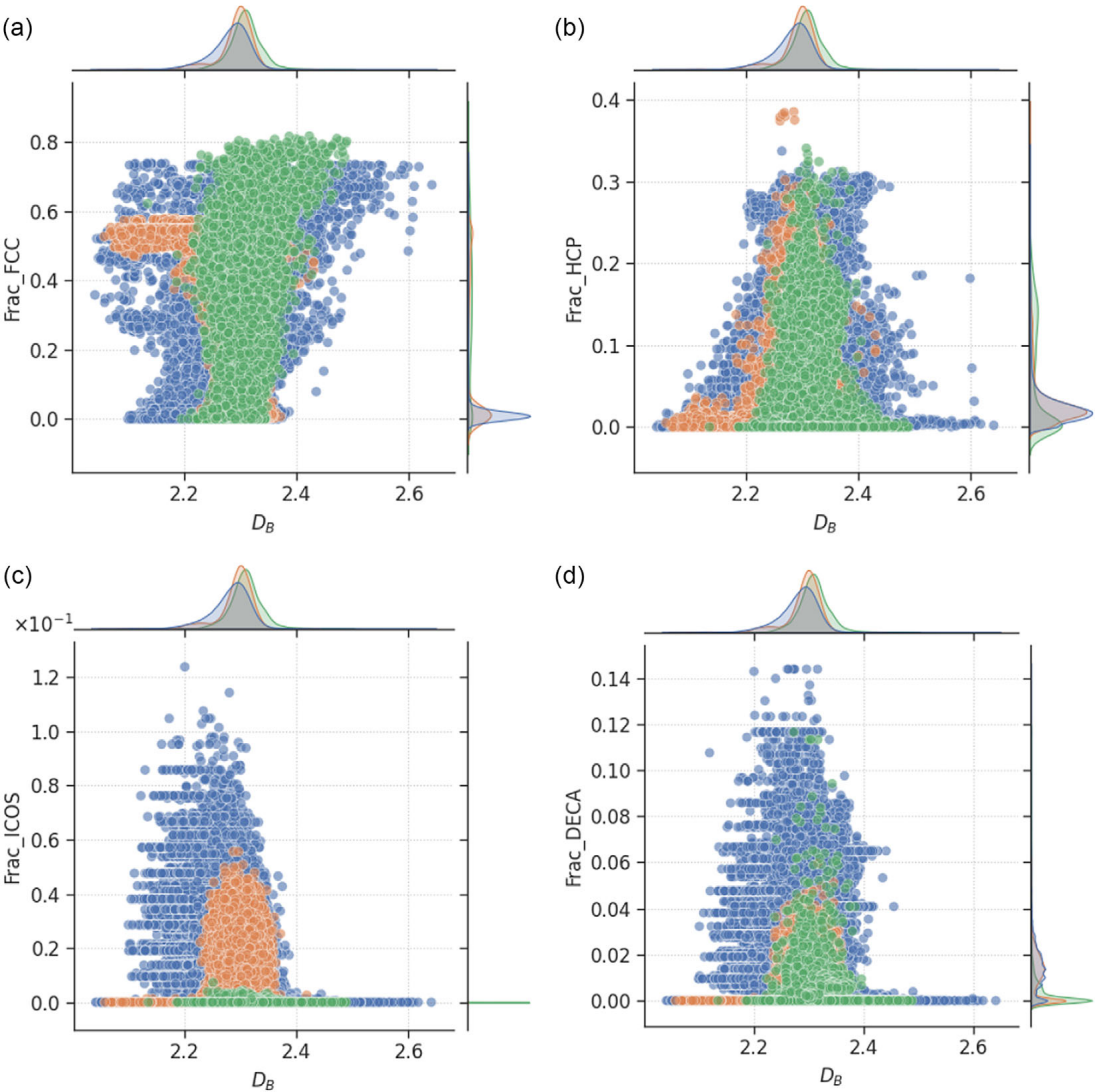
Scatter plot of box‐counting dimension and fraction of atoms with specific crystal structures, including a) face‐centered cubic, b) hexagonal close‐packed, c) icosahedral, and d) decahedral packing. The green, blue, and orange points correspond to monometallic, bimetallic, and trimetallic nanoparticles, respectively. The mutual information scores for the relationships are provided in Table 1, with higher values indicating greater dependencies of the feature on box‐counting dimension.

**Figure 10:**
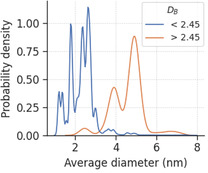
Probability density distribution of the average diameter for rough and smooth nanoparticles as defined by a box‐counting dimension threshold of 2.45, where the areas under the curves sum up to 1.

The relationships between nanoparticle $D_{B}$ and the fractions of atoms in certain crystal structures, including face‐centered cubic (FCC), hexagonal close‐packed (HCP), icosahedral, and decahedral packings, are shown in **Figure** **9**. In this case, the roughest nanoparticles tend to have high fraction of FCC atoms and low fractions of atoms in other crystal structures. The high fraction of any of these crystal structures indicates high crystallinity, although all component elements in this work prefer FCC packings.^[^ ^57^ ^]^ This is in agreement with the insight obtained from Section 2.2.4, and provides the manufacturers with an actionable insight to produce rougher nanoparticles, by choosing milder conditions such as lower temperature during the synthesis process to preserve the crystallinity order and minimize the introduction of structural disorder to the nanoparticles. However, the trend is not obvious for the TNP dataset, with the roughest nanoparticle having $D_{B}$ of less than 2.45, which is much lower than the other datasets. This can be attributed to bias in the sampling of the nanoparticle sizes in the dataset. Along with the findings in Section 2.2.3, this discovery suggests that the nanoparticles with $D_{B}$ above 2.45 are larger nanoparticles. **Figure** **10** confirms this hypothesis, as the average diameter distribution of the rougher nanoparticles peaks at a much larger value of 4.9 nm compared to 2.6 nm of the smoother nanoparticles, with minimal overlapping between the two distributions.

**Figure 11:**
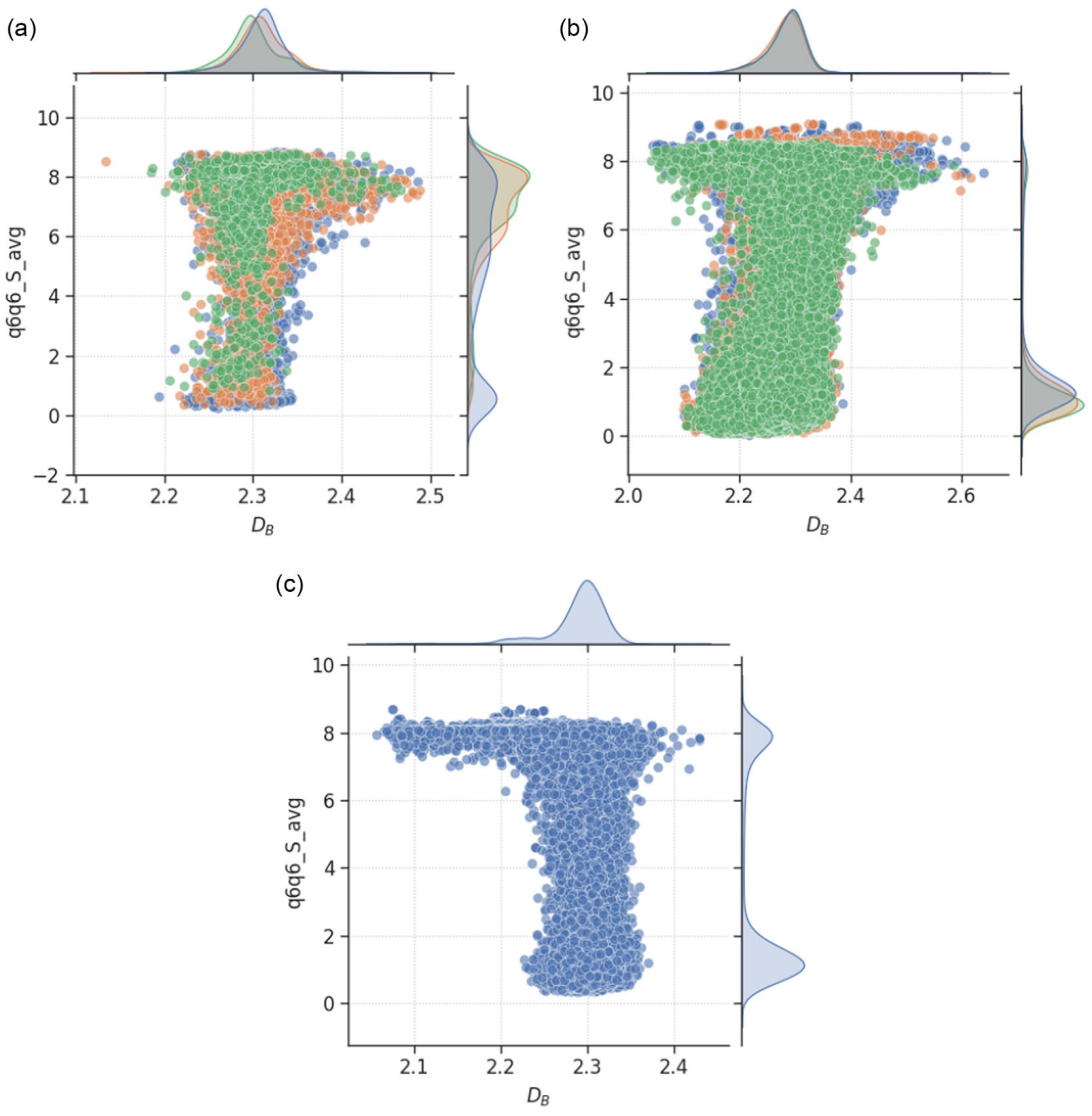
Scatter plot of box‐counting dimension and average number of bonded atoms for a) monometallic (with Au shown in blue, Pd in orange, and Pt in green), b) bimetallic (with AuPd shown in blue, AuPt in orange, and PdPt in green), and c) trimetallic (blue) nanoparticles. The mutual information scores for the relationships are provided in Table 1, with higher values indicating greater dependencies of the feature on box‐counting dimension.

The Steinhardt's bond‐order parameters, particularly $q_{6}$, provide useful information regarding the local crystallinity of nanoparticles.^[^ ^58^, ^59^, ^60^, ^61^ ^]^ Further explanations about the parameters are provided under Section 4.4. **Figure** **11** shows the relationship between the $D_{B}$ of nanoparticles and the average number of bonded neighbors of the surface atoms, which $D_{B}$ depends strongly upon based on the highest mutual information score among the investigated features according to Table 1. The plots for the bulk atoms and all atoms are included in the Supporting Information.

Across all nanoparticles, the average number of bonded neighbors for bulk, surface, and all (total) atoms showed bimodal distributions. The MNP datasets have higher proportion of more crystalline nanoparticles (with higher averages), followed by the TNP dataset, and the BNP datasets contain the least crystalline nanoparticles. This corresponds well with the simulation temperatures during the collection of the nanoparticle conformations shown in Figure 7. To ensure that the complete melting process is captured for all nanoparticles, the BNPs nanoparticles were sampled from higher temperatures compared to the TNP dataset, which is composed of only one elemental combination, namely, AuPdPt. Consequently, the bulk atoms of BNPs tend to have higher mobility, causing the nanoparticles to be more disordered and hence less crystalline.

It is clearly seen that the surface atoms of the roughest nanoparticles always have more bonded neighbors on average. This indicates greater surface crystallinity and complements the observations made thus far, that more ordered surfaces are actually higher in dimensionality, and hence rougher. Surface roughness is not the same as surface disorder. Therefore, we anticipate that these rough nanoparticles have relatively well‐structured surface packings with high number of bonded neighbors, such as the {100} facets (surface atoms with 8 neighbors), {111} facets (surface atoms with 9 neighbors), and {110} facets (surface atoms with either 7 or 11 neighbors). Given this, our next step of investigation focuses on the relationship between $D_{B}$ and the fractions of surface atoms lying on these facets.

As mentioned in Section 1, the computation of $D_{B}$ is sensitive to the orientation of the boxes, and hence is not rotationally nor translationally invariant. This is due to the discretization effect or quantization error resulting from the discrepancy between the 3D grid discretization and the actual space occupied by the object at some scales,^[^ ^46^, ^62^ ^]^ and is often ignored or addressed by brute‐force translations of the boxes,^[^ ^63^ ^]^ which was not done in this study due to the prohibitive amount of computational expenses required.

**Figure 12:**
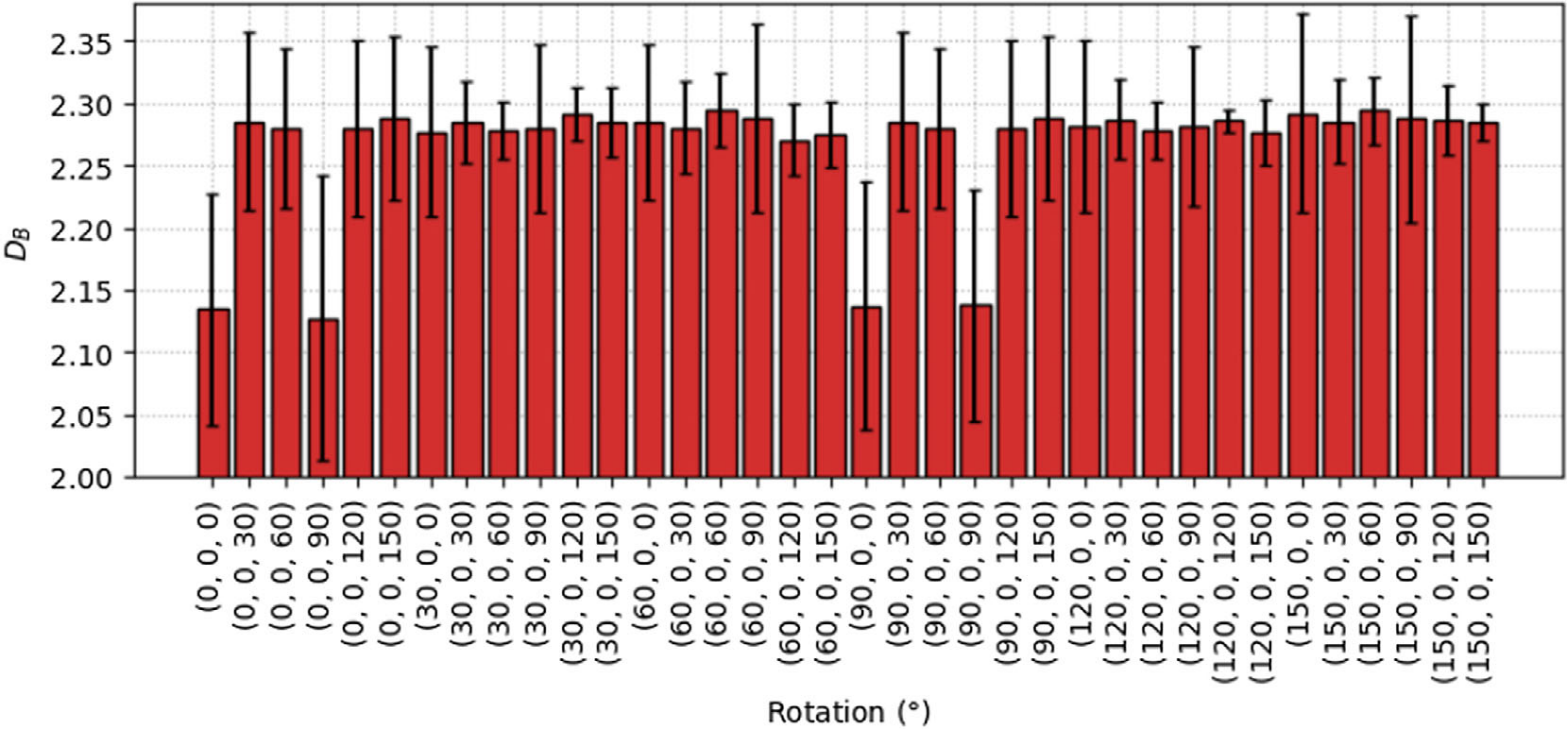
Box‐counting dimensions of the outlier nanoparticle rotated along the *x* and *y* axes.

**Figure 13:**
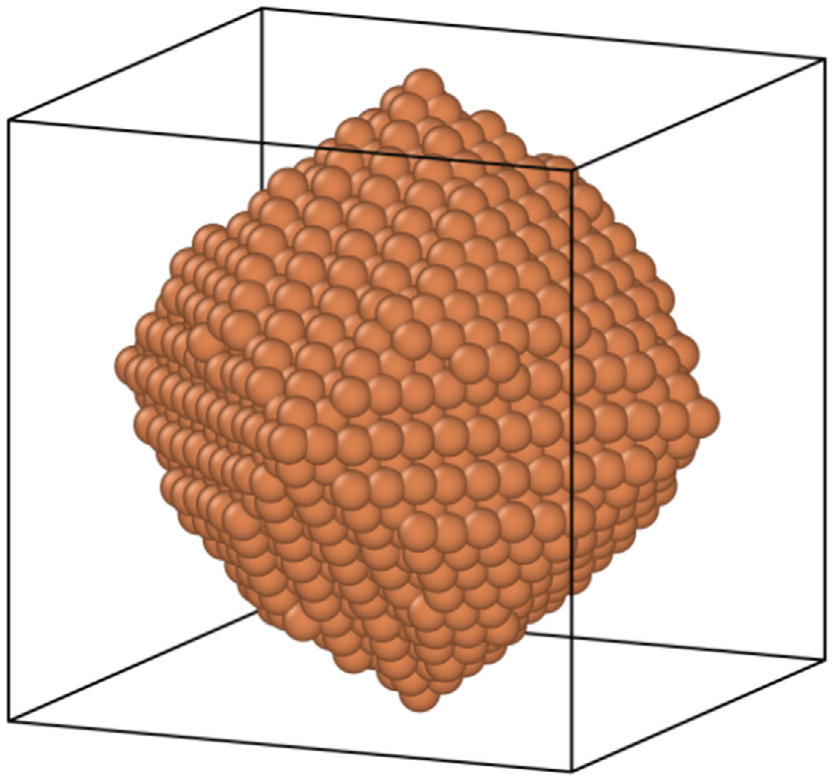
The orientation of the outlier palladium nanoparticle with respect to the simulation box. The outlier among the Pd nanoparticles with a lower *D*_*B*_ than other MNPs is found to be an ordered rhombic dodecahedron. While other ordered rhombic dodecahedra exists in the dataset, this configuration stands out as one that is particularly well aligned to the axes defining the simulation box.

**Figure** **12** shows the $D_{B}$ of an outlier nanoparticle that is rotated from its original orientation, shown in **Figure** **13**. In this case, the $D_{B}$ values are lowered to ≈2.13 whenever the rotation has a combination of 0° and 90° (which is approximately equal to the original orientation given the symmetricity of the rhombic dodecahedral nanoparticle), indicating that less boxes are counted as covering the surfaces when the nanoparticle is aligned in this particular manner. When the atom coordinates deviates from this orientation, the $D_{B}$ averages at ≈2.28. Even though this effect would be minimized in other nanoparticle samples where the coordinates are not perfectly aligned with the boxes, it is important to address the issues in future studies, potentially by randomly rotating the coordinates prior to the computation of $D_{B}$, or averaging the $D_{B}$ values from several rotated coordinates of the same coordinates.

#### 2.3.2 Surface Facets

**Figure 14:**
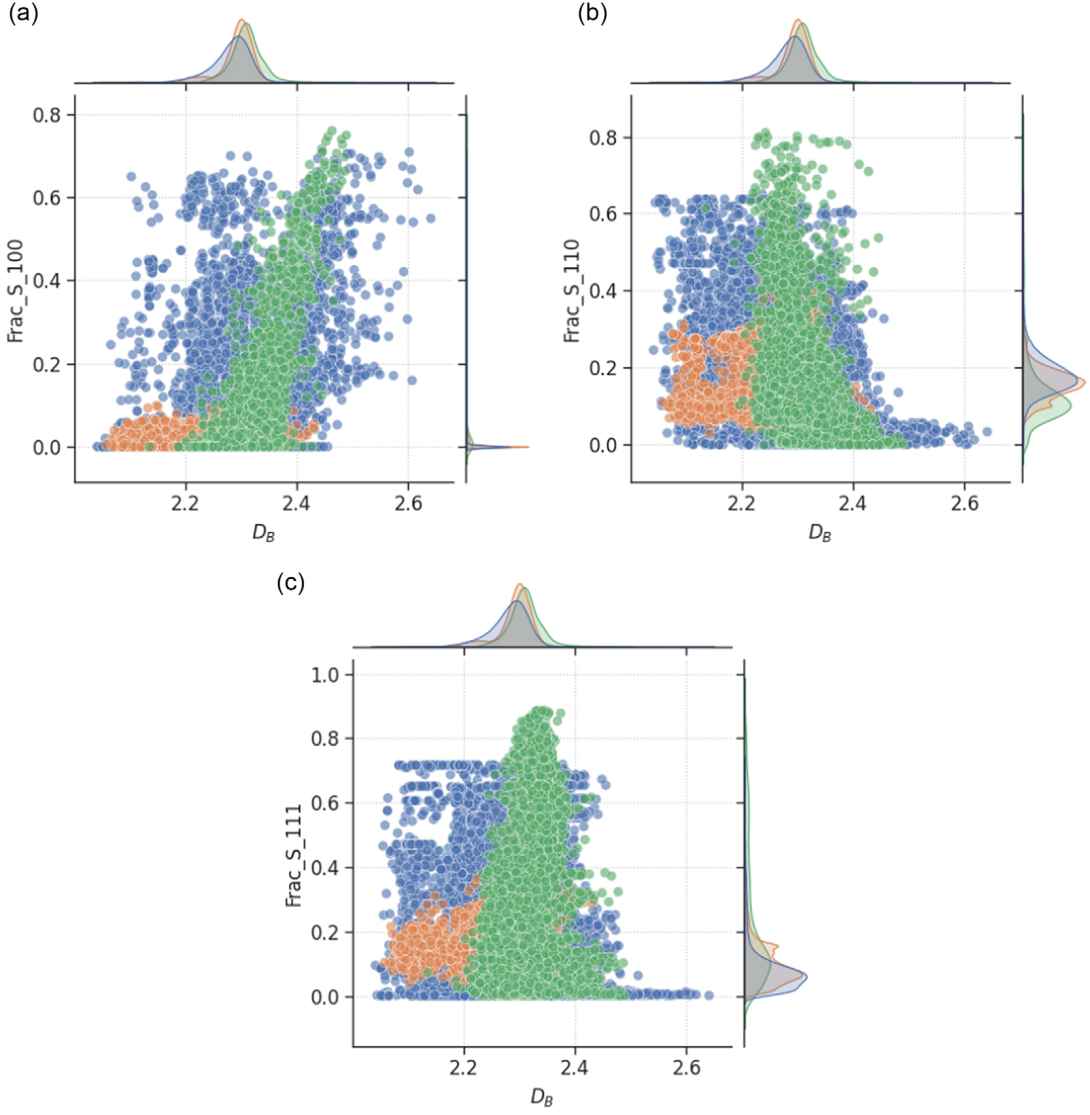
Scatter plot of box‐counting dimension and fraction of surface atoms lying on a) {100}, b) {110}, and c) {111} facets. The green, blue, and orange points correspond to monometallic, bimetallic, and trimetallic nanoparticles, respectively. The mutual information scores for the relationships are provided in Table 1, with higher values indicating greater dependencies of the feature on box‐counting dimension.

**Figure** **14** shows the relationship between $D_{B}$ and features related to surface facets. Roughest nanoparticles tend to have low fractions of surface atoms on the {110} and {111} facets, but high fraction of surface atoms on the {100} facet. This suggests that larger amount of concavities and convexities is captured by the box‐counting algorithm on the {100} facet compared to the others. As different types of surface facets are commonly associated with the catalytic activities toward different chemical reactions,^[^ ^64^ ^]^ this will be further investigated in the next section. The positive correlation between the fraction of surface atoms on the {100} facets and $D_{B}$ can be seen most clearly for Pt nanoparticles of the monometallic nanoparticles (see the element‐specific plots included in the Supporting Information). This is because the highest simulation temperature for the Pt dataset (623 K) is lower than Au and Pd (923 K). As Pt has higher melting point (2041 K for bulk) than Au (1337 K) and Pd (1828 K), Pt nanoparticles contain less structural disorder due to thermal contribution and retain higher proportion of structured surfaces. Given this observation, it is also anticipated that $D_{B}$ will show relatively higher dependency on atoms lying on lower surface curvature, which is a characteristic of {100} facets.

**Figure 15:**
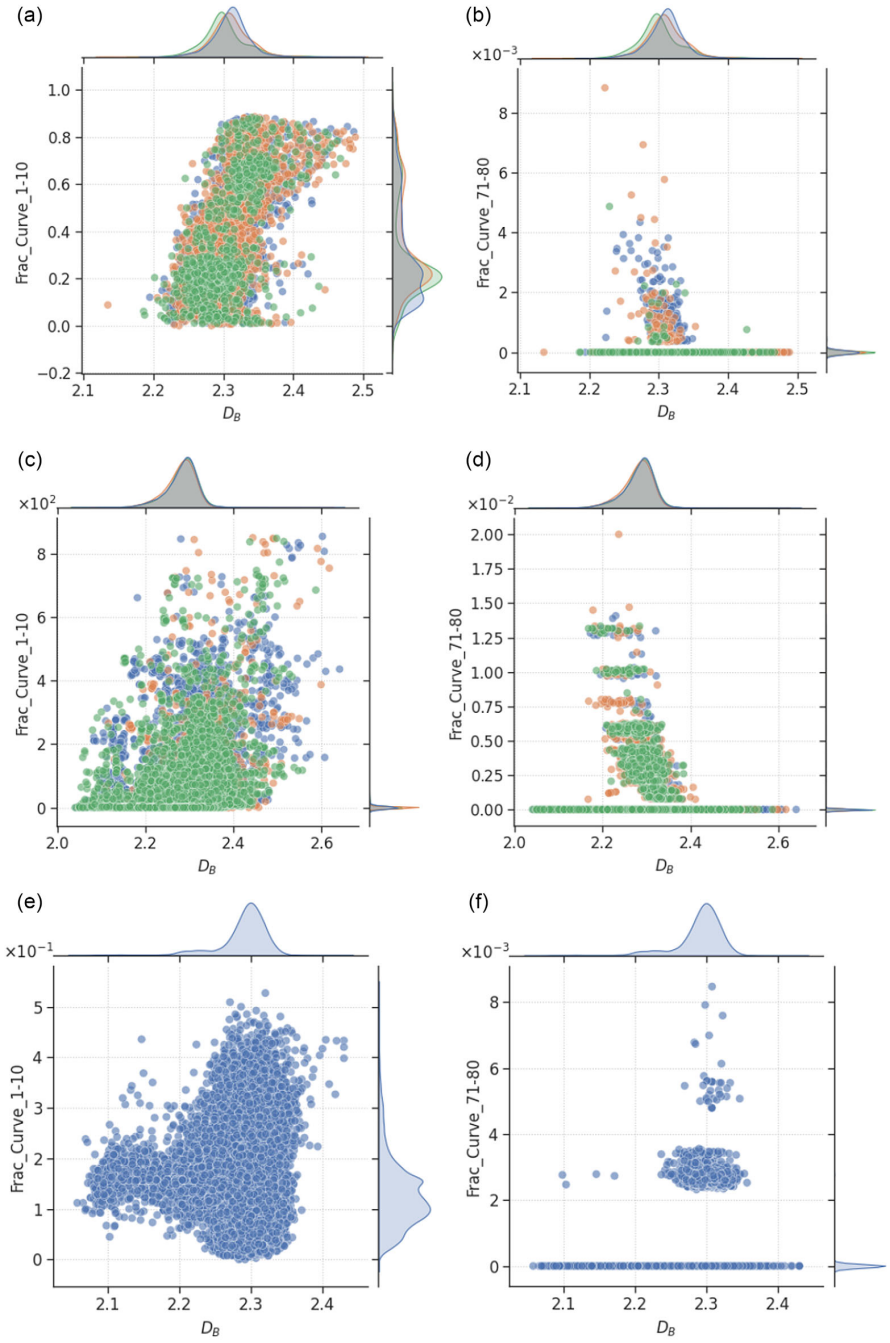
Scatter plot of box‐counting dimension and number of atoms lying on surface with a,c,e) 1–10° and b,d,f) 71–80° curvatures for monometallic (a,b: Au, blue; Pd, orange; Pt, green), bimetallic (c,d: AuPd, blue; AuPt, orange; PdPt, green), and trimetallic (e,f: blue) nanoparticles. The mutual information scores for the relationships are provided in Table 1, with higher values indicating greater dependencies of the feature on box‐counting dimension. Additional results are provided in the Supporting Information for comparison.

The relationships between $D_{B}$ and features related to surface curvature are shown in **Figure** **15**. In agreement with findings above, the plots indicate that the roughest nanoparticles tend to have higher fraction of atoms lying on surfaces with low curvatures, and vice versa. This suggests that nanoparticles with flatter surfaces should be made if achieving high roughness is the goal.

#### 2.3.3 Bond Length Statistics

Bond lengths and bond length statistics are more difficult to measure experimentally, but relationships between $D_{B}$ and these features are highly relevant for computational studies of metal nanoparticles.

**Figure 16:**
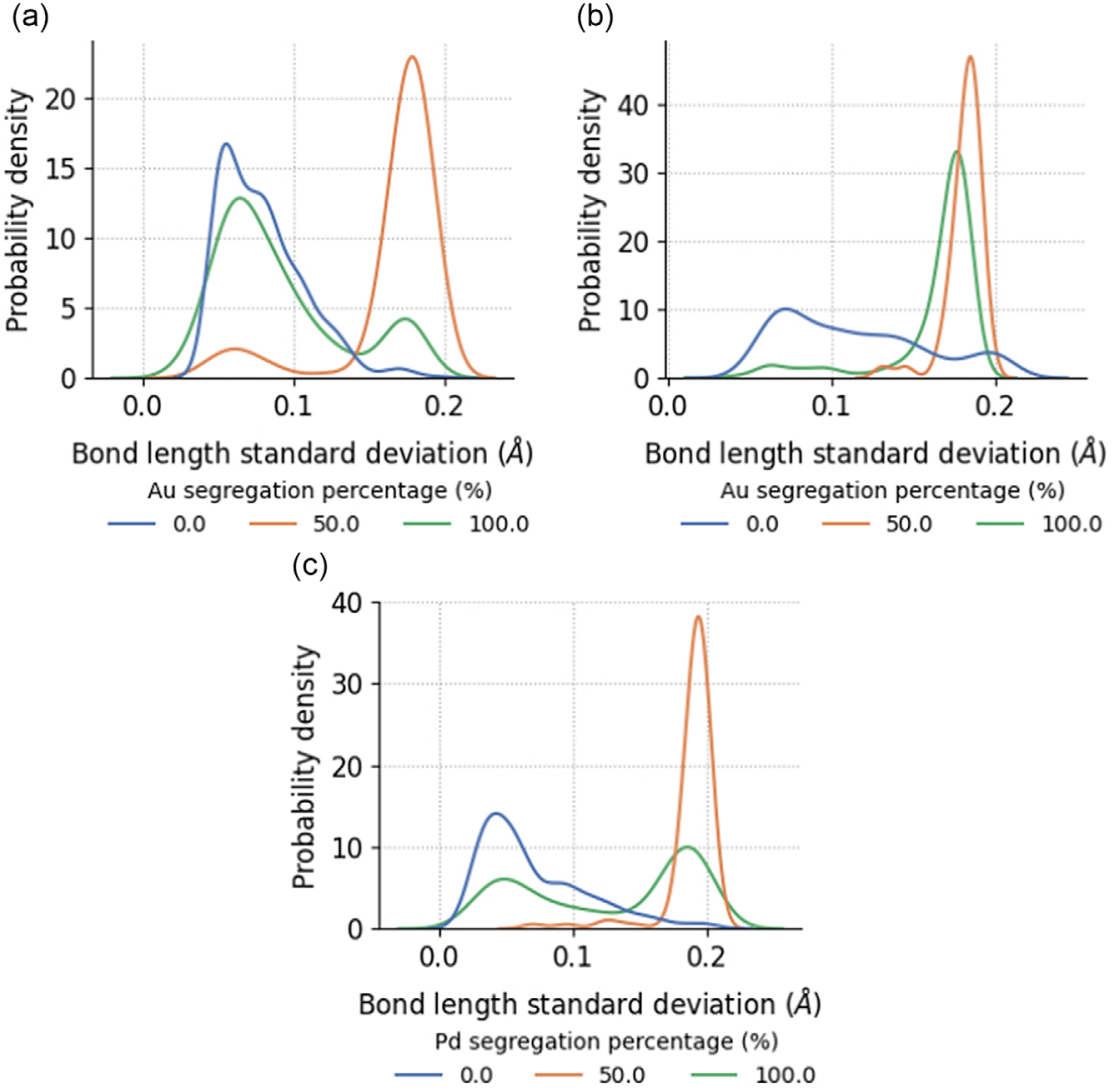
Probability density distributions of bond length standard deviations for a) AuPd, b) AuPt, and c) PdPt nanoparticles with different degrees of surface segregation, where the areas under the curves sum up to 1.

We find that the average bond lengths within the nanoparticles show slightly positive correlation with their $D_{B}$. This observation complements the findings in Section 2.2.1, as longer bond lengths further extend the distances between the surface atoms, allowing more concavities and convexities on the surface structure to be captured. The roughest nanoparticles also tend to have lower bond length standard deviations. The narrower distributions invite the following possibilities, all of which correspond well to the previous observations: 1) the rough nanoparticles are more ordered (see Section 2.2.4 and 2.3.1) as structural disorder inevitably introduces differences in bond lengths,^[^ ^65^ ^]^ 2) the rough nanoparticles are larger in size and have smaller proportion of atoms on the surface (see Figure 10), which are known to exhibit different bond lengths than the bulk atoms,^[^ ^66^, ^67^ ^]^ 3) in the case of multimetallic nanoparticles, the rougher nanoparticles with lower bond length standard deviations largely correspond to those that are surface segregated (as confirmed in **Figure** **16**), which have less bonds of varying lengths between different elements.

All figures from which the observations were made are included in the Supporting Information.

#### 2.3.4 Bond Angle Statistics

**Figure 17:**
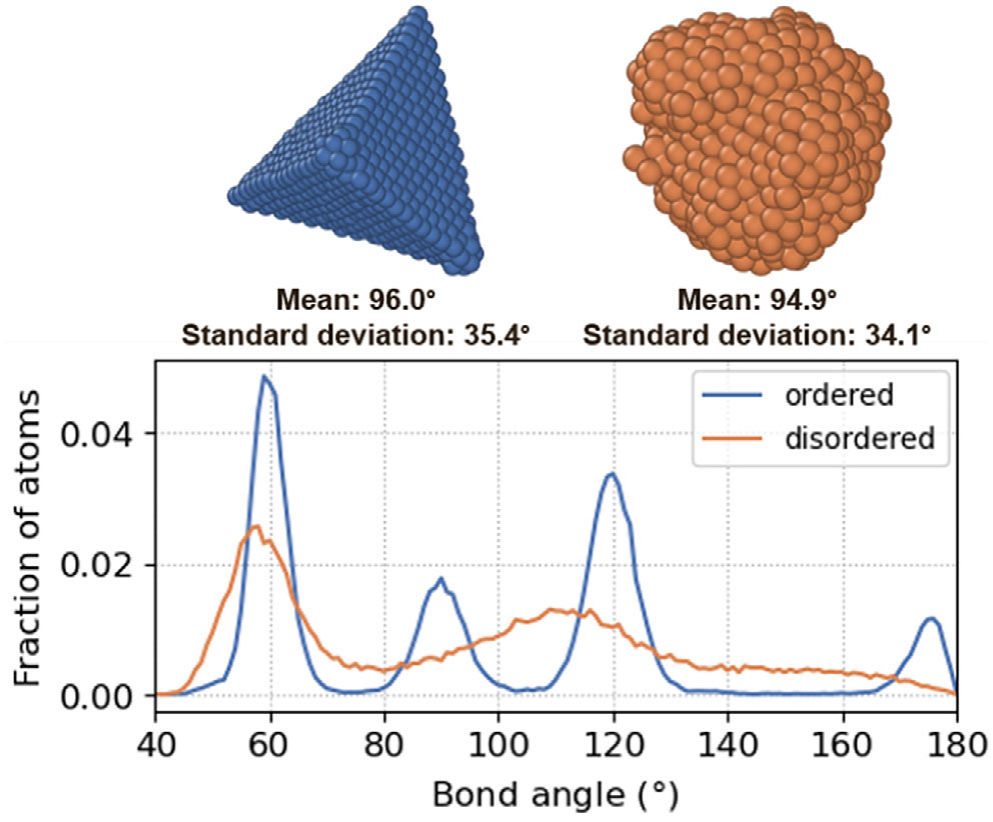
The bond angle distributions of Pd nanoparticles with higher and lower fractions of atoms in face‐centered cubic packings, denoted as ordered and disordered, respectively.

The nanoparticles with higher $D_{B}$ tend to have higher mean and standard deviation of nanoparticle bond angles. Given the observation from Figure 9a, these rough nanoparticles likely correspond to nanoparticles with higher fraction of FCC packings. This is confirmed by **Figure** **17**, where deviation from the FCC structures is found to result in lower average and standard deviation of bond angles. Compared to disordered nanoparticles with less FCC atoms, the bond angles of nanoparticles with more FCC atoms have higher average and wider distribution due to their distinctive peaks at 60°, 90°, 120°, and 180°, which merge together as the structures deviate from FCC packings. The apparent opposite trend seen among the TNPs (figure shown in Supporting Information) is likely due to the aforementioned size disparities between the TNP dataset and other datasets.

#### 2.3.5 Coordination Number

The roughest MNPs and BNPs with highest $D_{B}$ are more highly coordinated on average. The complementary statement (smoothest nanoparticles have lower average coordination numbers [CNs]) could be seen more obviously among the TNPs (figure included in the Supporting Information), particularly for the surface atoms. This indicates that the rougher metal nanoparticles have less surface defects, in contrast to the conventional understanding that surface defects result in rougher surfaces.^[^ ^68^, ^69^, ^70^ ^]^ Once again, this stresses the difference in the roughness captured by $D_{B}$ compared to the common concept of roughness uphold by the catalysis community, which could be more precisely phrased as disorder. $D_{B}$ quantifies roughness in terms of the amount of convexities and concavities on the surfaces, as opposed to simply changes in heights that the nanoscience community typically focuses on. One could understand it as the space‐filling capacity of the surfaces. The rougher nanoparticles have less surface defects because although their presence tends to induce differences in heights on the surfaces, the disorder in the surface structures also causes the gaps between the surface atoms to be filled with higher compactness. This is in agreement with the observation on the radial distribution functions of monometallic nanoparticles with different elements in Figure 3, where higher $D_{B}$ was obtained for nanoparticles with larger gaps between the atoms. This discrepancy in the understanding of roughness will likely contribute to the difficulties in connecting $D_{B}$ to catalysis. In light of these findings, we would like to encourage more careful usages of the term “roughness” by the catalysis community going forth.

The scatter plots of $D_{B}$ against the average bulk CN also reveal that the roughest nanoparticles tend to have an average CN of 12.0; the deviation from this value (bulk CN for FCC and HCP packing) correlates to decreasing surface roughness. This again indicates that the amount of disorder within a nanoparticle is inversely proportional to its surface roughness, leading to the same conclusion that the preservation of structural order is crucial in the synthesis of rough nanoparticles.

### 2.4 Relating Dimensionality to Catalytic Activity

The utility of this exercise comes in relating the nanoparticle surface roughness (as quantified by $D_{B}$) to the known catalytic activities exhibited by nanoparticles enclosed by surface facets with {100}, {110}, and {111} crystallographic orientations, which dominate most nanoparticles under reaction conditions due to their lower surface energies compared to surfaces with higher Miller indices.^[^ ^71^ ^]^

Beginning with a set of simulated Pd nanoparticles with specific shapes, we computed their $D_{B}$ to determine the facet‐dependent values. The shapes of the nanoparticles are chosen to cover the range of common types of surface facets, including octahedron (OT) enclosed by {111} facets, cube (CU) enclosed by {100} facets, and rhombic dodecahedron (RD) enclosed by {110} facets. Given that each of these nanoparticles is completely enclosed by the same surface facet, the $D_{B}$ of the whole nanoparticle converges to the $D_{B}$ of the surface facet as the nanoparticle size increases.

**Figure 18:**
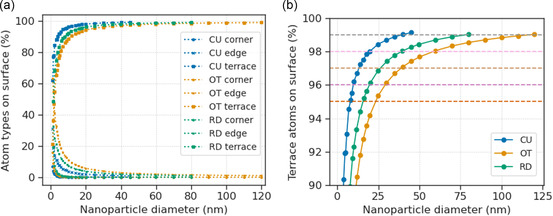
The a) different types of atoms and b) terrace atoms on nanoparticle surface as nanoparticle diameter increases.

Ideally, the nanoparticles should be large enough such that the impact of corners and edges of the nanoparticles on the surface $D_{B}$ is minimized. **Figure** **18** shows the change in the percentages of the surface atom types as the size of the nanoparticles increases (from 3 to 100 nm). The type of surface atom is determined by their CNs, with the rules listed below: For OT nanoparticle, atoms with CN of 4 are regarded as corners, CN of 7 are on the edges, and CN of 9 are on the facets or terraces. The corners, edges, and terraces on the CU nanoparticle have CNs of 3, 5, and 8, respectively. The corner atoms of the RD nanoparticle have CNs of either 3 or 4, edge atoms have CNs of either 5 or 10, and terrace atoms have CNs of either 7 or 11. The largest nanoparticles that were processed in this work are those with diameters of 119.8, 45.1, and 80.1 nm for OT, CU, and RD, respectively, the surfaces of which are covered by >99% of terrace atoms.

**Figure 19:**
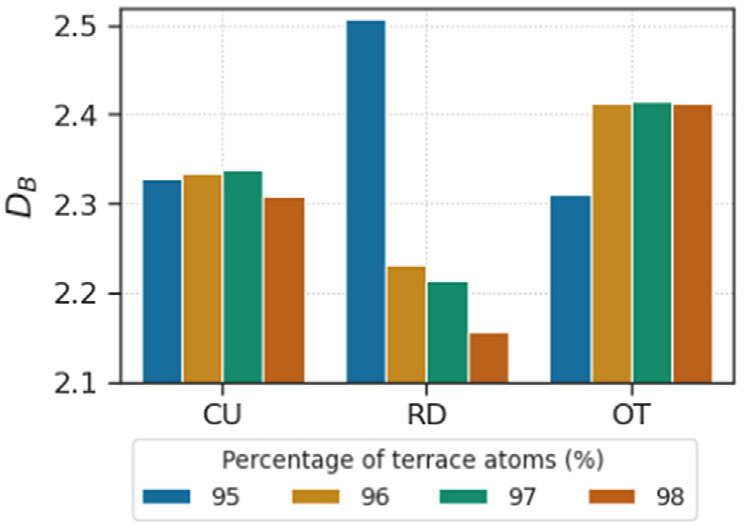
Box‐counting dimensions of nanoparticles with different surface facets.

**Figure** **19** shows the $D_{B}$ of the Pd nanoparticles with >95% terrace atoms on their surfaces, which follows the trend of OT > CU > RD (equivalent to {111} > {100} > {110}). It is observed that the $D_{B}$ of RD nanoparticles is more sensitive toward nanoparticle size than CU and OT. Although the smaller nanoparticles (with ≈95% surface atoms being terrace atoms) do not follow the same trend, greater weight should be placed on the nanoparticles with a larger percentage of surface atoms being terrace as the influence of corner and edge atoms are minimized in these cases.

Upon comparisons with the literature related to the catalytic activities of Pd facets, we discovered both agreement and discrepancies with the trend observed in this study. We highlight that the exact opposite trend of reactivities ({110} > {100} > {111}) has been reported by Kim et al. for the oxidative addition of aryl bromide^[^ ^72^ ^]^ and Shenoy et al. for the binding of chlorine^[^ ^73^ ^]^ to Pd nanoparticles. Hori has also reported that the {110} facets are more catalytically active for the reduction of carbon dioxide than {111} facets,^[^ ^74^ ^]^ and the superior reactivity of the {100} facet over {111} facet was reported for peroxide decomposition^[^ ^75^ ^]^ and nitroaniline reduction.^[^ ^76^ ^]^ Although the direction of the relationship is reversed (higher $D_{B}$ means lower catalytic activity), $D_{B}$ could be used to explain the activities in these cases. These findings allude to the potential of $D_{B}$ in rationalizing the trends of catalytic activities observed in particular types of nanoparticles toward certain reactions.

On the other hand, a different trend of {100} > {110} > {111} has also been reported for the dehydrogenation of dodecahydro‐*N*‐ethylcarbazole,^[^ ^77^ ^]^ and hydrogen evolution reaction.^[^ ^78^ ^]^ In the case of catalysis of formic acid oxidation reaction by Pd nanoparticles, both trends of {100} > {110} > {111} and {100} > {111} > {110} have been reported, depending on the solvent used in the experiments (H_2_SO_4_ for former,^[^ ^79^ ^]^ and HClO_4_ for latter^[^ ^80^ ^]^). The trend observed in our case matches the trend for Pt nanoparticles.^[^ ^80^ ^]^

Nonetheless, we note that the trend observed here is the same as the surface energy^[^ ^81^ ^]^ and thermodynamic stability^[^ ^82^ ^]^ of each facet, and correlates with their number of vacant chemical bonds, which are 3, 4, and 5 for {111}, {100}, and {110}, respectively.^[^ ^83^ ^]^ The matching trends between these measures, which are all relevant for catalysis, point to the potential of using $D_{B}$ as a cheaper alternative to provide similar information. This means that $D_{B}$ can indirectly inform researchers of the catalytic activities of nanoparticles, and help to rationalize trends between facets and reactions.

## 3 Conclusion

In this work, we investigated the relationships between the fractal dimension of metal nanoparticle surfaces as estimated by their box‐counting dimensions with experimentally and computationally relevant nanoparticle structural features on a set of simulated nanoparticles data consisting of gold, palladium, and platinum. Nanoparticle alloying was found to have a surface smoothening effect, with the surface segregation increasing the dimensionality of the nanoparticle surfaces, regardless of the relative concentration of the elements. Rougher nanoparticles tend to have the following characteristics: 1) They are mostly sampled from lower temperature. 2) They are larger in size. 3) They have higher fractions of atoms with FCC crystal structures, hence average bulk CNs that is more closely centered at 12. 4) They have higher fractions of surface atoms on {100} facets, hence more atoms on flatter surfaces, and surface atoms with more bonded neighbors. 5) They have longer bond lengths on average and narrower bond length distributions. 6) They have larger bond angles and wider bond angle distributions.

Based on these results, we propose that rough nanoparticles can be synthesized by employing conditions and methods that aid in the preservation of order in their structures by inducing the growth and stabilization of certain crystallographic facets or allowing reorganization of atoms on disordered surfaces into more ordered structures. Such approaches include keeping the processing conditions at lower temperature, lowering the rate of addition of metal precursors, employing surfactants and capping agents,^[^ ^84^ ^]^ leveraging template synthesis,^[^ ^85^ ^]^ and postsynthesis mild annealing.^[^ ^86^, ^87^, ^88^ ^]^

The characteristics of rough nanoparticles unanimously suggest that preserving order in nanoparticle structures results in higher surface dimensionality. Hence, we suggest that an alternative way of measuring roughness at the nanoscale is to define roughness in terms of the area occupied by the nonoverlapping outer surface of the atomic spheres. This complexity or roughness of the surface can be quantitatively captured by the box‐counting dimension.

The differences in the trends of reactivities are expected as catalysts are often facet‐selective,^[^ ^64^ ^]^ and heterogeneous catalysis via metal nanoparticles is often affected by multiple factors other than surface area of catalysts, including underlying structure below nanoparticle surfaces,^[^ ^83^ ^]^ steric hindrances affecting the active site accessibility,^[^ ^89^, ^90^, ^91^ ^]^ and type of chemical species involved in the reactions both in terms of reactants and nanoparticle surface compositions,^[^ ^92^ ^]^ which affects the electronic structures,^[^ ^91^ ^]^ reaction kinetics,^[^ ^93^ ^]^ and adsorption–desorption properties.^[^ ^94^ ^]^ The present investigation on the relationship between $D_{B}$ and the catalytic activity of nanoparticles has specifically focused on the facet types, and the dependencies of $D_{B}$ on the other factors have yet to be explored. Additionally, the experimental catalytic activities are often measured over prolonged period, during which surface reconstruction often takes place,^[^ ^91^ ^]^ while the dynamic properties of the surface structure were not taken into account here. These might have contributed to the differences in the trend observed here compared to the literature.

The connection between the fractal box‐counting dimension and the catalytic activities of the low‐index surface facets toward certain industrially relevant chemical reactions was also investigated. While no direct relationship was established, the box‐counting dimension was shown to indirectly relate to features important to catalysis, such as surface energy, thermodynamic stability, and chemical bond vacancy of the facets. Given the importance of metal nanoparticle surfaces dynamics in the performance of catalysts, these findings will assist researchers in designing rougher nanoparticles, and help to develop a more detailed understanding about the surface complexity. Quantifying surface roughness using the box‐counting dimension may also be applicable to other fields, such as photothermal conversion,^[^ ^95^ ^]^ chemical sensing,^[^ ^96^ ^]^ and nanoparticle dispersion rheology.^[^ ^97^ ^]^

The present study has yet to be linked to experimental data which is sparse and to our knowledge, there exists no readily available experimental datasets quantifying the surface roughness of individual metal nanoparticles. We suggest that such connection could be made by methods that can produce 3D spatial coordinates from experimental data such as (Hybrid) Reverse Monte Carlo.^[^ ^98^, ^99^ ^]^ These methods are capable of generating plausible atomic coordinates by minimizing cost functions based on fittings to coordinates‐derived physical quantities obtained from experimental data, and have been successfully applied to small nanoparticles.^[^ ^100^, ^101^, ^102^ ^]^ A wide array of experimental data can be used including those obtained from techniques such as X‐Ray diffraction, extended X‐Ray absorption fine structure data, nuclear magnetic resonance, and fluctuation electron microscopy.^[^ ^103^ ^]^ To experimentally explore the correlation of the surface fractal dimension of metal nanoparticles with their structural features and catalytic activities, we suggest characterization of a set of metal nanoparticles using these techniques, and then using Reverse Monte Carlo methods to generate atomistic models of the nanoparticle structures. This would allow the subsequent quantification of their surface roughness by the computation of $D_{B}$, and enable more direct links between the surface roughness and features or properties of interest to be made.

## 4 Experimental Section

4.1

4.1.1

##### Datasets

The datasets consist of simulated nanoparticles generated from classical molecular dynamics with embedded atom interatomic potentials,^[^ ^104^ ^]^ encompassing monometallic to trimetallic combinations of Au, Pd, and Pt, resulting in a total of seven datasets, designated as Au, Pd, Pt, AuPd (merged with PdAu), AuPt (merged with PtAu), PdPt (merged with PtPd), and AuPdPt. The process for generating the MNP datasets is explained in ref. [105]. The details for the generation of the BNP and TNP datasets are provided alongside the datasets on the data repository websites. The datasets are diverse, and each structure is unique, including ordered crystalline nanoparticles, disordered noncrystalline nanoparticles, and polycrystalline nanoparticles including twinned nanoparticles. **Table** **2** describes the data sizes and the range of nanoparticle sizes of the original (and reduced in the case of BNPs) datasets. The range of the number of atoms and diameters remained the same after the redundancy filtering reduction for BNP datasets.

**Table 2:** Descriptions of the original datasets, with citations to the public repository (ref.).

Dataset	Number of features	Original of number samples	Final number of samples	Percentage of reduction [%]	Minimum and maximum atom number	Minimum and maximum diameter [nm]	Ref.
Au	182	4000	4000	0.0	236–14 277	1.7–7.8	[33]
Pd	182	4000	4000	0.0	137–16 262	1.4–7.5	[34]
Pt	182	1300	1300	0.0	54–15 837	1.5–7.6	[35]
AuPd	922	145 103	47 623	67.2	93–4631	1.1–5.1	[36]
AuPt	922	162 439	53 445	67.1	93–4631	1.1–5.1	[37]
PdAu	922	145 064	48 087	66.9	105–4631	1.2–5.0	[38]
PdPt	922	151 216	48 785	67.7	105–4631	1.2–5.0	[39]
PtAu	922	162 770	54 006	66.8	105–4631	1.2–5.1	[40]
PtPd	922	150 781	48 943	67.5	105–4631	1.2–5.0	[41]
AuPdPt	1958	48 136	48 136	0.0	603–959	2.3–2.9	[42]

##### Box‐Counting Dimension

The $D_{B}$ of each metal nanoparticle is computed from their atomic coordinates using an open source Python package, namely, Sphractal.^[^ ^29^ ^]^ Assuming that each atom is a perfect sphere, the surface of the given nanoparticle is represented as a mathematically exact surface formed by the nonoverlapping part of the spherical atomic surfaces, and the roughness measure is computed using a box‐counting approach.^[^ ^28^ ^]^ Readers are directed to the publicly available notebook at https://github.com/Jon‐Ting/metal‐nanoparticle‐surface‐fractal‐dimension/blob/main/sphractal‐parameter‐tuning.ipynb for the details of the parameter‐tuning process and the justifications for each relevant parameter. The parameters that we used for $D_{B}$ calculation are: *radType* = “atomic,” *radMult* = 1.2, *calcBL* = False, *findSurfAlg* = “alphaShape,” *alphaMult* = 2.0, *bulkCN *= 12, *trimLen* = True, *confLvl*=95, *rmInSurf* = True, *minSample* = 5, *voxelSurf* = False, *minLenMult* = 0.15, *maxLenMult* = 2.00, *numBoxLen* = 10, and *bufferDist* = 5.0. For the shape‐specific Pd nanoparticles (generated using the Atomic Simulation Environment package^[^ ^106^ ^]^ with a lattice constant of 3.89^[^ ^107^ ^]^), a simpler criterion based on the number of neighbors is used instead of the more time‐consuming alpha shape algorithm^[^ ^108^ ^]^ to identify the surface atoms due to the large size of the nanoparticles. The differences in the parameters used are: *findSurfAlg* = “numNeigh,” *radMult* = 1.2 for CU, 1.1 for RD and OT, *bulkCN* = 14 for CU, 12 for RD and OT.

##### Radial Distribution Function

The radial distribution function is calculated using Network Characterization Package,^[^ ^109^ ^]^ which is a publicly available software that performs structural analysis for atomistic objects. The relevant parameters involved are radii cutoff of 3.5 for all three types of metals (Au, Pd, Pt), buffer distance of 10 Å from simulation box boundaries for each axis, reduced density computed as number of particles divided by volume of simulation box (30 × 30 × 30 Å^3^), spacing interval of 0.01 Å, and 600 sampling points. All code used could be found at https://github.com/jon‐ting/metal‐nanoparticle‐surface‐fractal‐dimension.

##### Steinhardt's Parameters

It was proposed that the local orientational structure of a given atom *i* can be characterized^[^ ^58^, ^110^, ^111^ ^]^ by Equation (2)

(2) $${\bar{q}}_{lm}(i)=\frac{1}{n(i)}\sum_{j=1}^{n(i)}Y_{lm}(r_{ij})$$

where n(i) is the number of neighbors of atom *i*; $r_{ij}$ is the orientation vector pointing from atom *i* to atom *j*; and $Y_{lm}(r_{ij})$ is the spherical harmonic corresponding to $r_{ij}$.

As shown in Equation (3), a rotationally invariant $q_{l}(i)$ can then be constructed to measure the local bond order around atom *i*. The parameter with *l* = 6 are known to be particularly sensitive to the degree of crystallinity but not differences between possible crystal structures,^[^ ^112^ ^]^ such as FCC, HCP, and random close‐packed.^[^ ^59^, ^110^ ^]^

(3) $$q_{lm}(i)=\frac{{\bar{q}}_{lm}(i)}{{\left(\sum_{m=-l}^{l}\left|{\bar{q}}_{lm}(i)\right|\right)}^{\frac{1}{2}}}$$

An atom *i* is considered bonded to its neighboring atom *j* if their local orientational orders match coherently, as defined by $\sum_{m=-6}^{6}q_{6m}(i)q_{6m}{\left(j\right)}^{*}>q_{thresh}$, with 0.7 being the consensus value for $q_{thresh}$. Atoms with a number of bonded neighbors ($n_{b}(i)$) that is close to the bulk CN (12 in the case of FCC crystals) are considered crystalline.

##### Statistical Analysis

Mutual information^[^ ^113^ ^]^ is computed in this work to capture the dependency between the features of interest and $D_{B}$, instead of the more commonly employed Pearson's correlation coefficient. This is because most of the bivariate relationships are nonlinear, but the latter is suited to linear dependencies. Mutual information of 0 means statistical independence and higher values indicate greater dependency. The mutual information scores are calculated in a nonparametric manner based on entropy estimation from *k*‐nearest neighbors distances^[^ ^114^, ^115^, ^116^ ^]^ via the publicly available scikit‐learn package.^[^ ^117^ ^]^ The significance of each relationship is assessed by the *p*‐value determined using the maximum entropy model^[^ ^118^ ^]^ (Equation (4)), at significance level of 0.05. Bonferroni correction was applied to adjust the significance level to control the overall probability of a type I error by dividing the significance level by the total number of hypotheses to be tested for each dataset.

(4) $$p=e^{-N\times MI}$$

where *N* is the number of samples and *MI* is the mutual information between a pair of variables.

## Conflict of Interest

The authors declare no conflict of interest.

## Supporting information

Supplementary Material

## Data Availability

All datasets used in this study are available in a public repository, including DOIs. Since a large number of individual sets are used, a list of all sets in provided in Table 2, with references containing links to each set.
