# Supplementary material for: Fractal Characterization of Simulated Metal Nanocatalysts in 3D
Source: Small Sci. 2024 Jul 9;4(10):2400123. doi: 10.1002/smsc.202400123 (PMC11935087; doi:10.1002/smsc.202400123)
Supplement: Supplementary file 1 — Supplementary Material [file SMSC-4-2400123-s001.pdf]

SUPPORTING INFORMATION
Fractal Characterisation of Simulated Metal Nanoparticle Surfaces for Catalytic Applications

Jonathan Yik Chang Ting *[1], George Opletal[2], and Amanda S. Barnard[1]

[1]*School of Computing, Australian National University, Acton 2601, Australia*
[2]*Modelling and Simulations, Private Bag 10, , Commonwealth Scientific and Industrial Research Organisation, 3169 Melbourne, VIC, Australia.*

This document contains details related to the sample reduction procedure for the bimetallic nanoparticles data sets in Section S1, the meaning fo all features investigated in Section S2, and scatter plots generated from the exploration of the relationships between the box-counting dimension (D_B) and structural features of nanoparticles in Section S3.

S1 Sample Reduction

The samples in the bimetallic nanoparticles data sets are reduced by identifying and removing the redundant conformations. The conformation redundancy is assessed based on the following metrics:

1. root-mean-square deviation (calculated in Euclidean distance) of the current conformation from the previous conformation (with a threshold of 1.0),

2. Wasserstein distance [1] (with a threshold of 0.0002), Hellinger distance [2] (with a threshold of 0.15), and Jensen-Shannon divergence [3] of the radial distribution function (RDF) of the current conformation from the RDF of the previous conformation (with a threshold of 0.02),

3. p-values (with thresholds of 0.05) from 2-sample Anderson-Darling test, 2-sample Kolmogorov-Smirnov test, and 2-sample Cramer-von Mises test, which test the hypothesis that there is no significant difference between the radial distribution functions of the current and previous conformations, and

4. difference in the box-counting dimensions of the current and previous conformations (with a threshold of 0.15).

The sample reduction workflow is illustrated in Figure S1. The threshold of each metric is decided by observing the changes of the metric during the melting simulation trajectories for a few chosen nanoparticles.

*Corresponding author: jonathan.ting@anu.edu.au

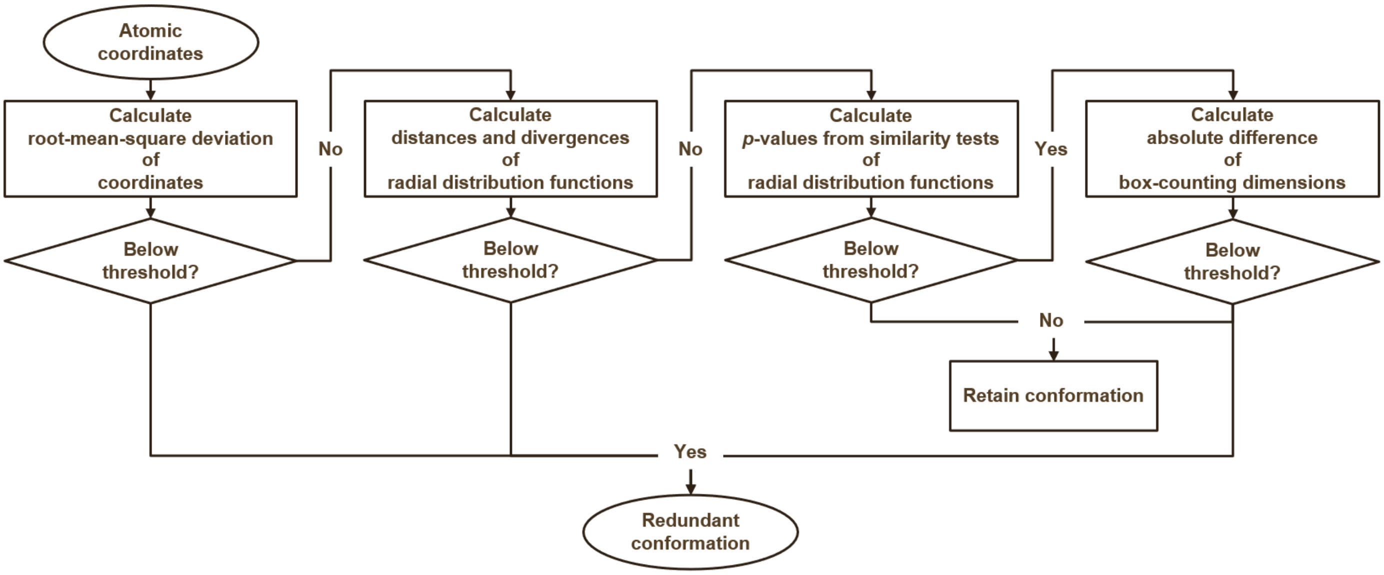

Figure S1: Overview of the workflow of the identification of redundant nanoparticle conformations.

S2 Feature Interpretation

The meanings of the features investigated in this work are listed under Table S1.

Table S1: Interpretation of nanoparticle features investigated.

Feature	Meaning
T	Simulation temperature (K)
D_avg	Average nanoparticle diameter (nm)
Frac_FCC	Fraction of atoms in face centred cubic lattice
Frac_HCP	Fraction of atoms in hexagonal close-packed lattice
Frac_ICOS	Fraction of atoms in icosahedral lattice
Frac_DECA	Fraction of atoms in decahedral lattice
q6q6_S_avg	Average number of bonded neighbours for all surface atoms
q6q6_B_avg	Average number of bonded neighbours for all bulk atoms
q6q6_T_avg	Average number of bonded neighbours for all atoms
Frac_S_100	Fraction of surface atoms located on {100} surfaces
Frac_S_110	Fraction of surface atoms located on {110} surfaces
Frac_S_111	Fraction of surface atoms located on {111} surfaces
Frac_Curve_X-Y	Fraction of surface atoms lying on surface with curvature angle of X-Y°
MM_BL_avg	Average bond length for all atoms (Å)
MM_BL_std	Standard deviation of bond lengths for all atoms (Å)
MM_BA1_avg	Average bond angle for all atoms (°)
MM_BA1_std	Standard deviation of bond angles for all atoms (°)
MM_SCN_avg	Average coordination number of all surface atoms
MM_BCN_avg	Average coordination number of all bulk atoms
MM_TCN_avg	Average coordination number of all atoms
N_atom_total	Total number of atoms
Volume	Nanoparticle volume computed from bulk density assumption (m^3)

S3 Relationship with Structural Features

This section contains the scatter plots generated from the exploration of the relationships between D_B and structural features of nanoparticles. This includes:

- Crystal Structures: Figures S2, S3, and S4 show the element-specific relationships between D_B and the fractions of atoms in the nanoparticles with particular crystal structures, including face-centred cubic, hexagonal close-packed, icosahedral, and decahedral packings.
- Steinhardt's Parameters: Figure S5 shows the relationship between D_B and the average number of bonded atoms for the bulk and all atoms of the nanoparticles.
- Surface Facets: Figures S6, S7, and S8 show the element-specific relationships between D_B and the fractions of surface atoms lying on surfaces with particular crystallographic orientations, including the {100}, {110}, and {111} facets.
- Surface Curvature: Figures S9, S10, and S11 show the element-specific relationships between D_B and the fractions of surface atoms lying on surfaces with particular range of curvature.
- Bond Length: Figure S12 shows the relationships between D_B and features related to bond length statistics.
- Bond Angle: Figure S13 shows the relationships between D_B and features related to bond angle statistics.
- Coordination Number: Figure S14 shows the relationship between D_B and the average coordination number of the nanoparticles.
- Total Number of Atoms: Figure S15 shows the relationship between D_B and the total number of atoms in the nanoparticles.
- Nanoparticle Volume: Figure S16 shows the relationship between D_B and the nanoparticle volume, which is computed from the number of atoms based on the assumption that the nanoparticles have the bulk density, which are 19320, 12023, and 21450 kg/m^3 for gold (Au), palladium (Pd), and platinum (Pt), respectively. For multimetallic nanoparticles, the element-specific volumes are summed up to give the final volume.

References

[1] L. V. Kantorovich, *https://doi.org/10.1287/mnsc.6.4.366* **1960**, *6* 366.

[2] R. Beran, *The Annals of Statistics* **1977**, *5* 445.

[3] B. Fuglede, F. Topsøe, *IEEE International Symposium on Information Theory - Proceedings* **2004**, 31.

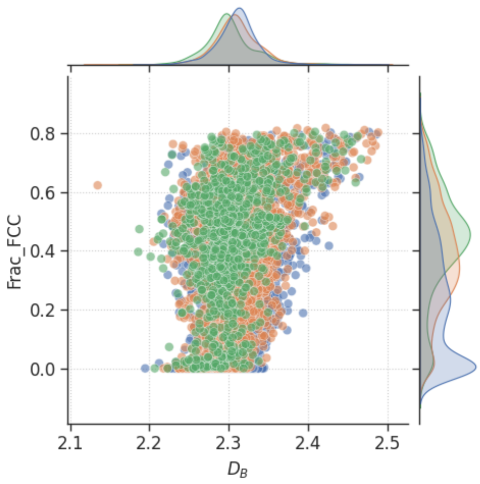

(a)

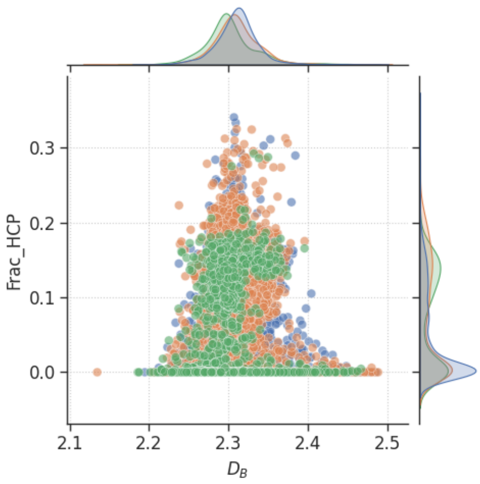

(b)

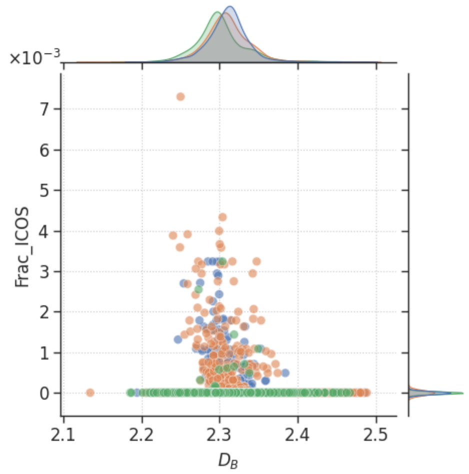

(c)

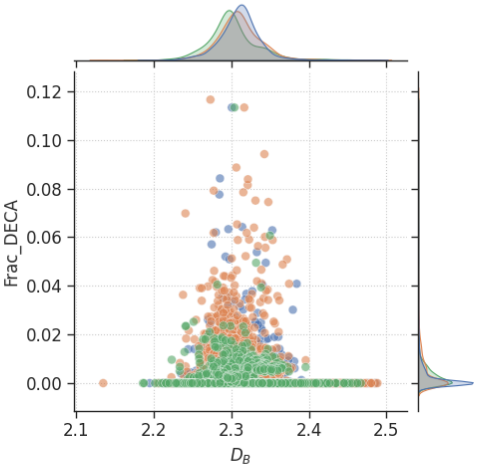

(d)

Figure S2: Scatter plots of box-counting dimension and fraction of atoms with specific crystal structures, including (a) face-centered cubic, (b) hexagonal close-packed, (c) icosahedral, and (d) decahedral packing, for monometallic nanoparticles. The blue, orange, and green points correspond to Au, Pd, and Pt nanoparticles, respectively. The mutual information scores for the relationships provided in main text Table 1, with higher values indicating greater Scatter plots of dependencies of the features on box-counting dimension.

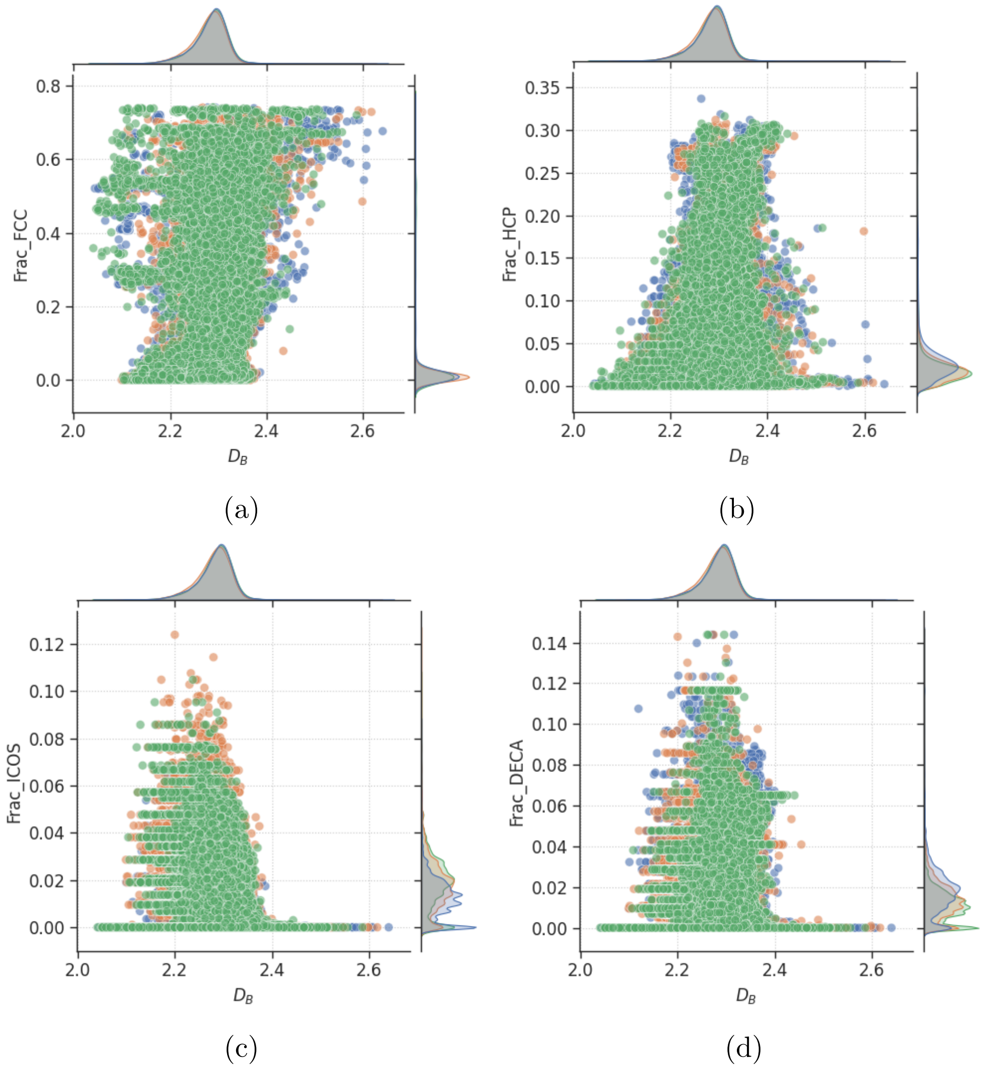

Figure S3: Scatter plots of box-counting dimension and fraction of atoms with specific crystal structures, including (a) face-centered cubic, (b) hexagonal close-packed, (c) icosahedral, and (d) decahedral packing, for bimetallic nanoparticles. The blue, orange, and green points correspond to AuPd, AuPt, and PdPt nanoparticles, respectively. The mutual information scores for the relationships provided in main text Table 1, with higher values indicating greater Scatter plots of dependencies of the features on box-counting dimension.

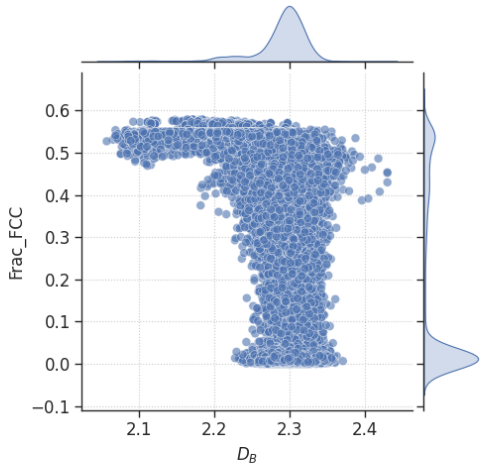

(a)

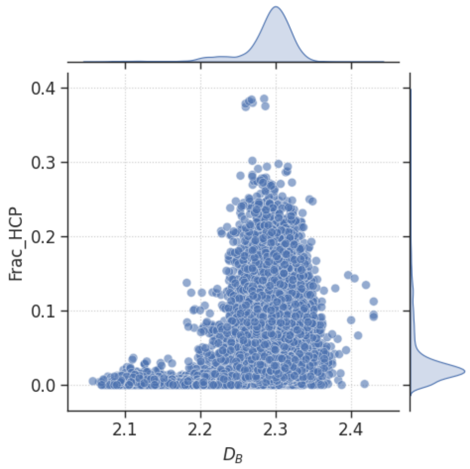

(b)

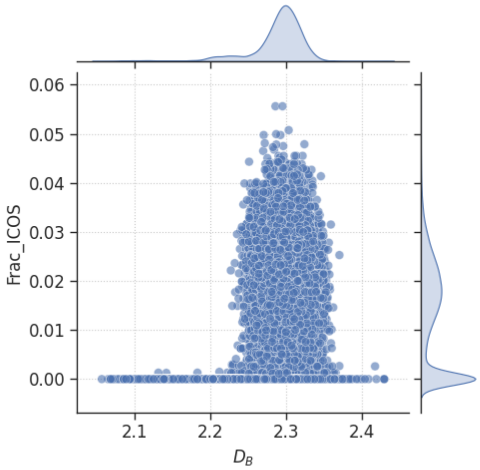

(c)

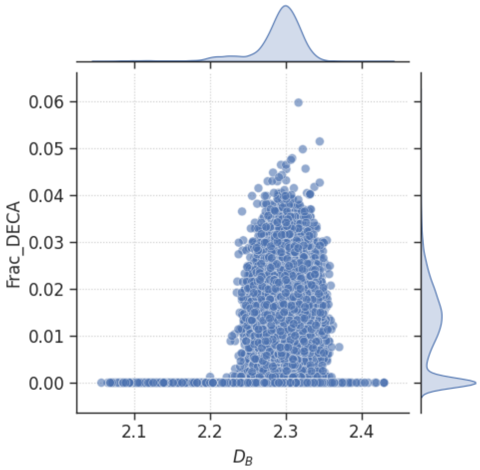

(d)

Figure S4: Scatter plots of box-counting dimension and fraction of atoms with specific crystal structures, including (a) face-centered cubic, (b) hexagonal close-packed, (c) icosahedral, and (d) decahedral packing, for trimetallic nanoparticles. The mutual information scores for the relationships provided in main text Table 1, with higher values indicating greater Scatter plots of dependencies of the features on box-counting dimension.

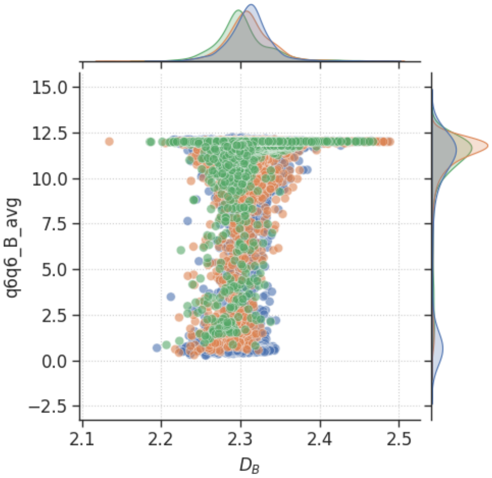

(a)

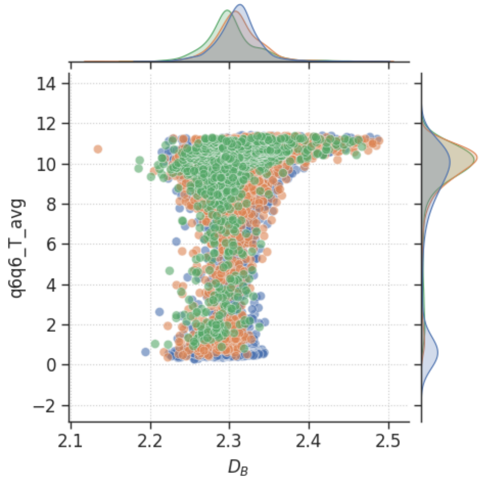

(b)

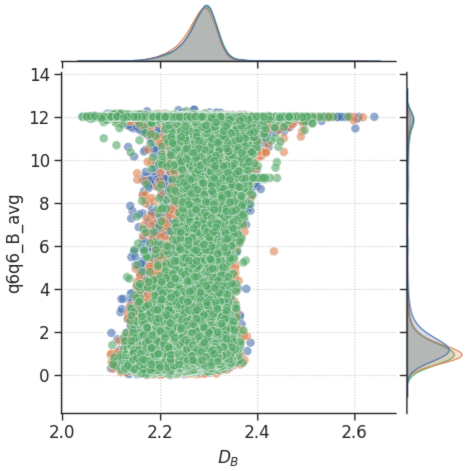

(c)

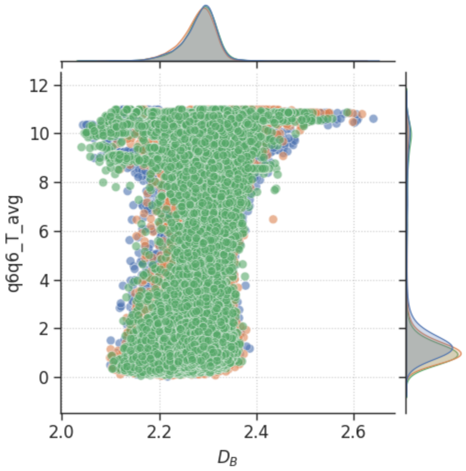

(d)

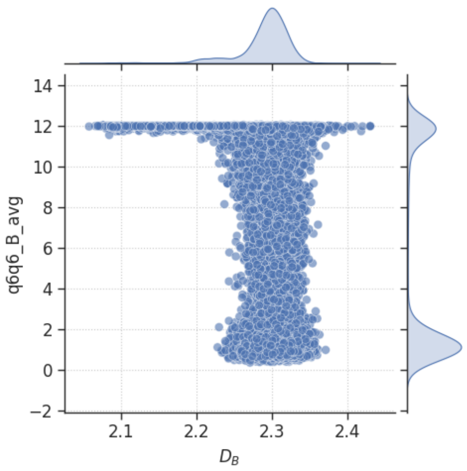

(e)

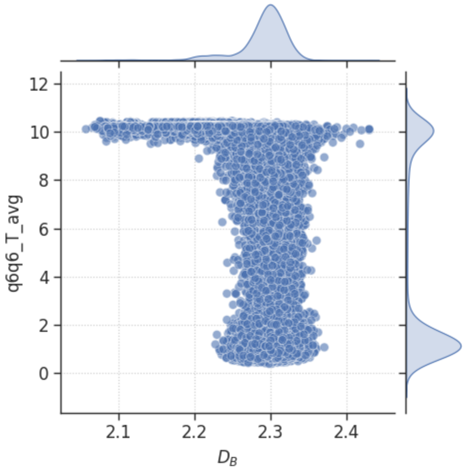

(f)

Figure S5: Scatter plots of box-counting dimension and average number of bonded atoms for bulk (left) and all (right) atoms of monometallic (upper, Au = blue, Pd = orange, Pt = green), bimetallic (middle, AuPd = blue, AuPt = orange, PdPt = green), and trimetallic (lower, blue) nanoparticles. The mutual information scores for the relationships are provided in main text Table 1, with higher values indicating greater Scatter plots of dependencies of the features on box-counting dimension.

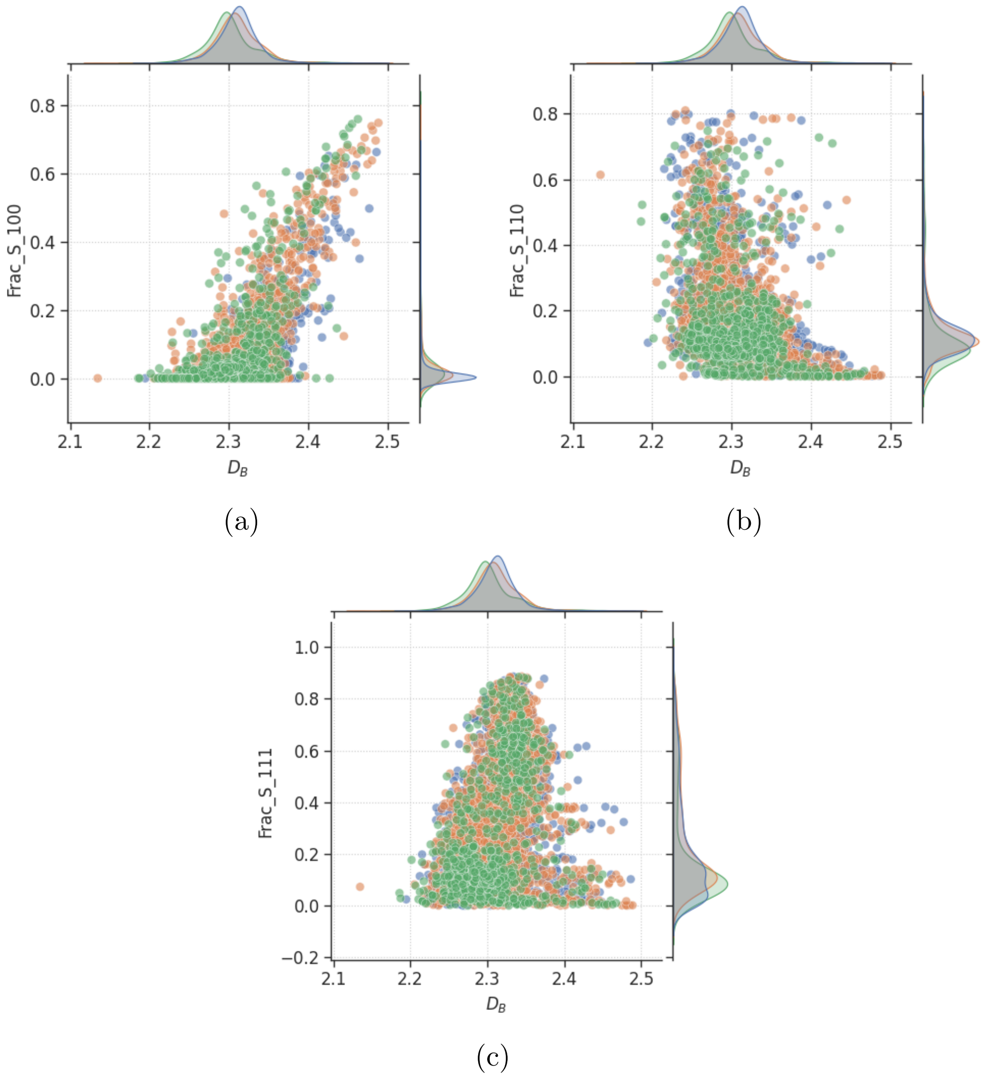

Figure S6: Scatter plots of box-counting dimension and fraction of surface atoms lying on (a) {100}, (b) {110}, and (c) {111} facets, for monometallic nanoparticles. The blue, orange, and green points correspond to Au, Pd, and Pt nanoparticles, respectively. The mutual information scores for the relationships are provided in main text Table 1, with higher values indicating greater Scatter plots of dependencies of the features on box-counting dimension.

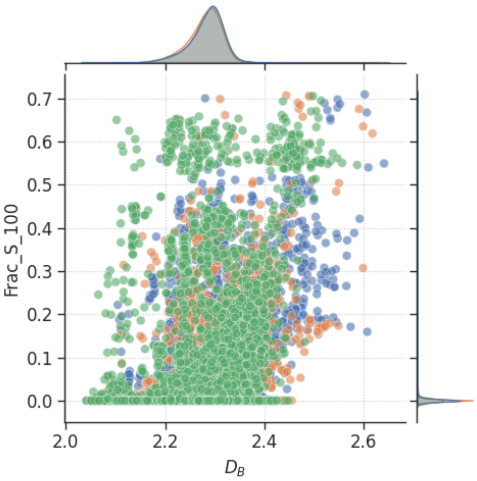

(a)

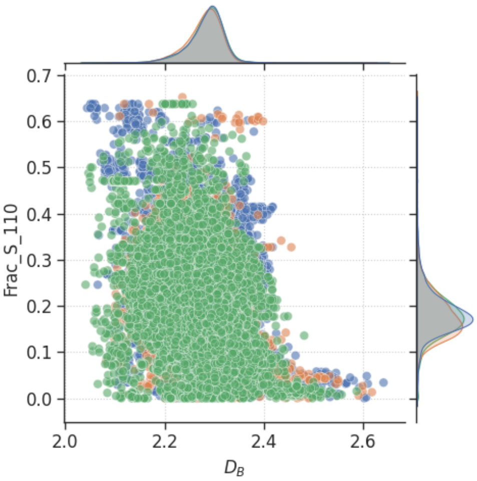

(b)

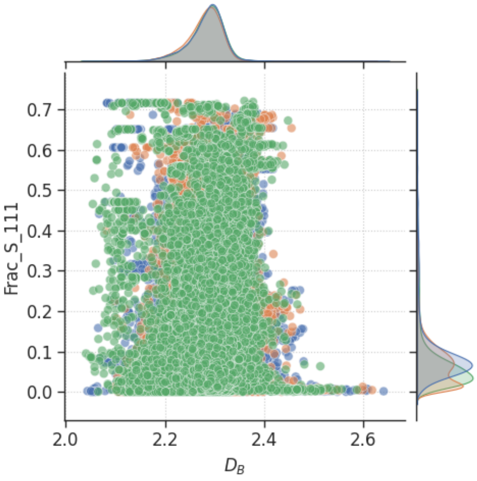

(c)

Figure S7: Scatter plots of box-counting dimension and fraction of surface atoms lying on (a) {100}, (b) {110}, and (c) {111} facets, for bimetallic nanoparticles. The blue, orange, and green points correspond to AuPd, AuPt, and PdPt nanoparticles, respectively. The mutual information scores for the relationships are provided in main text Table 1, with higher values indicating greater Scatter plots of dependencies of the features on box-counting dimension.

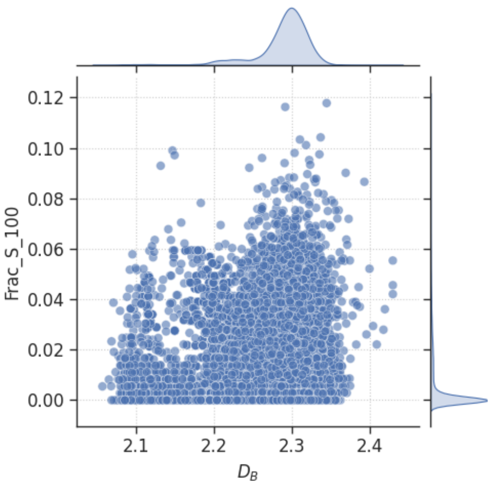

(a)

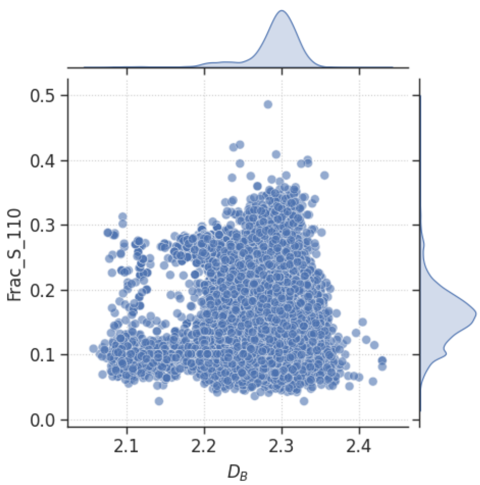

(b)

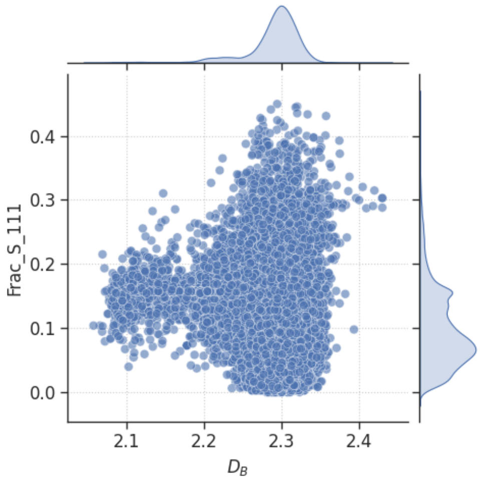

(c)

Figure S8: Scatter plots of box-counting dimension and fraction of surface atoms lying on (a) {100}, (b) {110}, and (c) {111} facets, for trimetallic nanoparticles. The mutual information scores for the relationships are provided in main text Table 1, with higher values indicating greater Scatter plots of dependencies of the features on box-counting dimension.

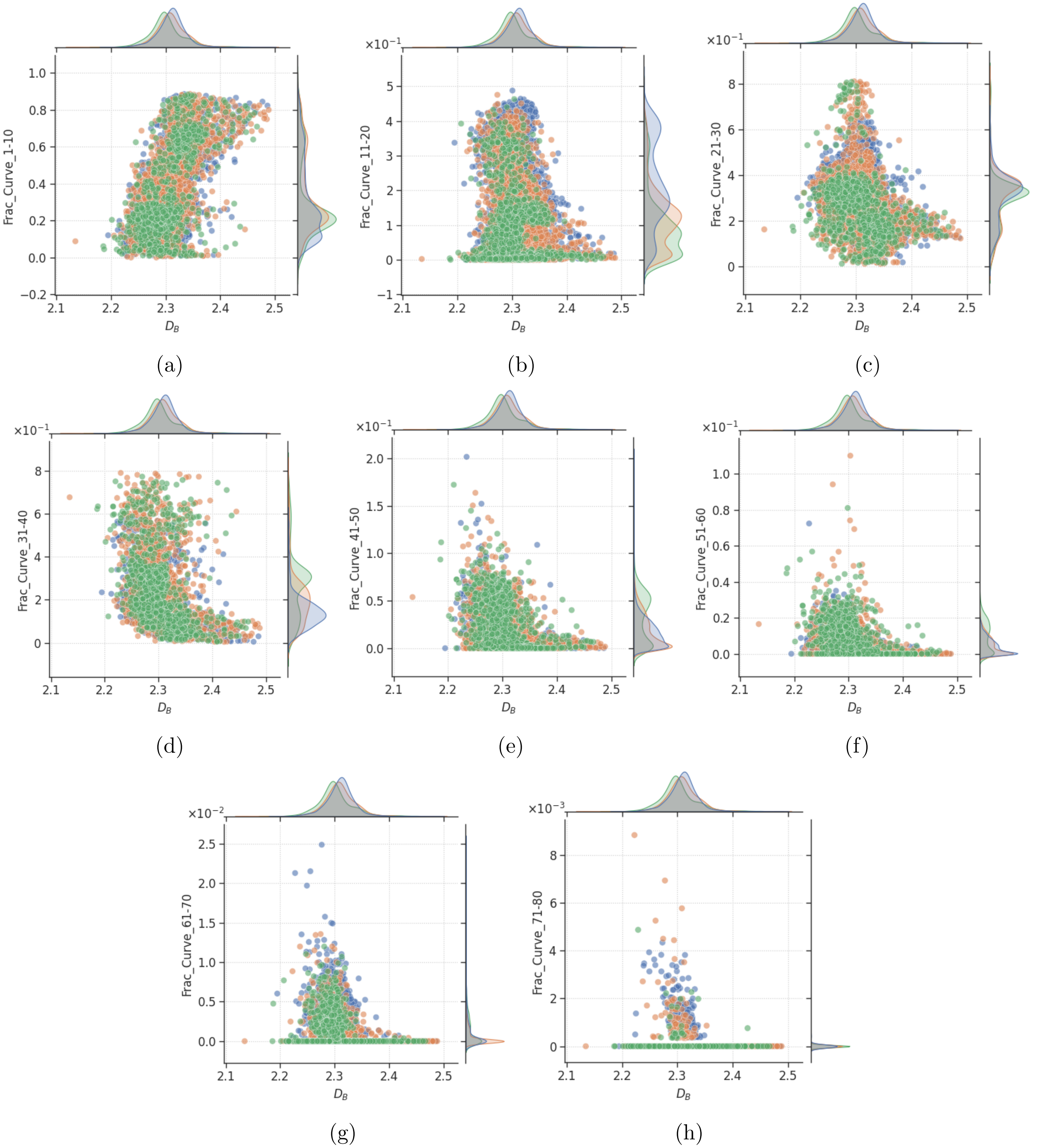

Figure S9: Scatter plots of box-counting dimension and fraction of surface atoms lying on surface with curvatures ranging from (a) 1° to 10°, (b) 11° to 20°, (c) 21° to 30°, (d) 31° to 40°, (e) 41° to 50°, (f) 51° to 60°, (g) 61° to 70°, and (h) 71° to 80° for monometallic nanoparticles. The blue, orange, and green points correspond to Au, Pd, and Pt nanoparticles, respectively. The mutual information scores for the relationships are provided in main text Table 1, with higher values indicating greater Scatter plots of dependencies of the features on box-counting dimension.

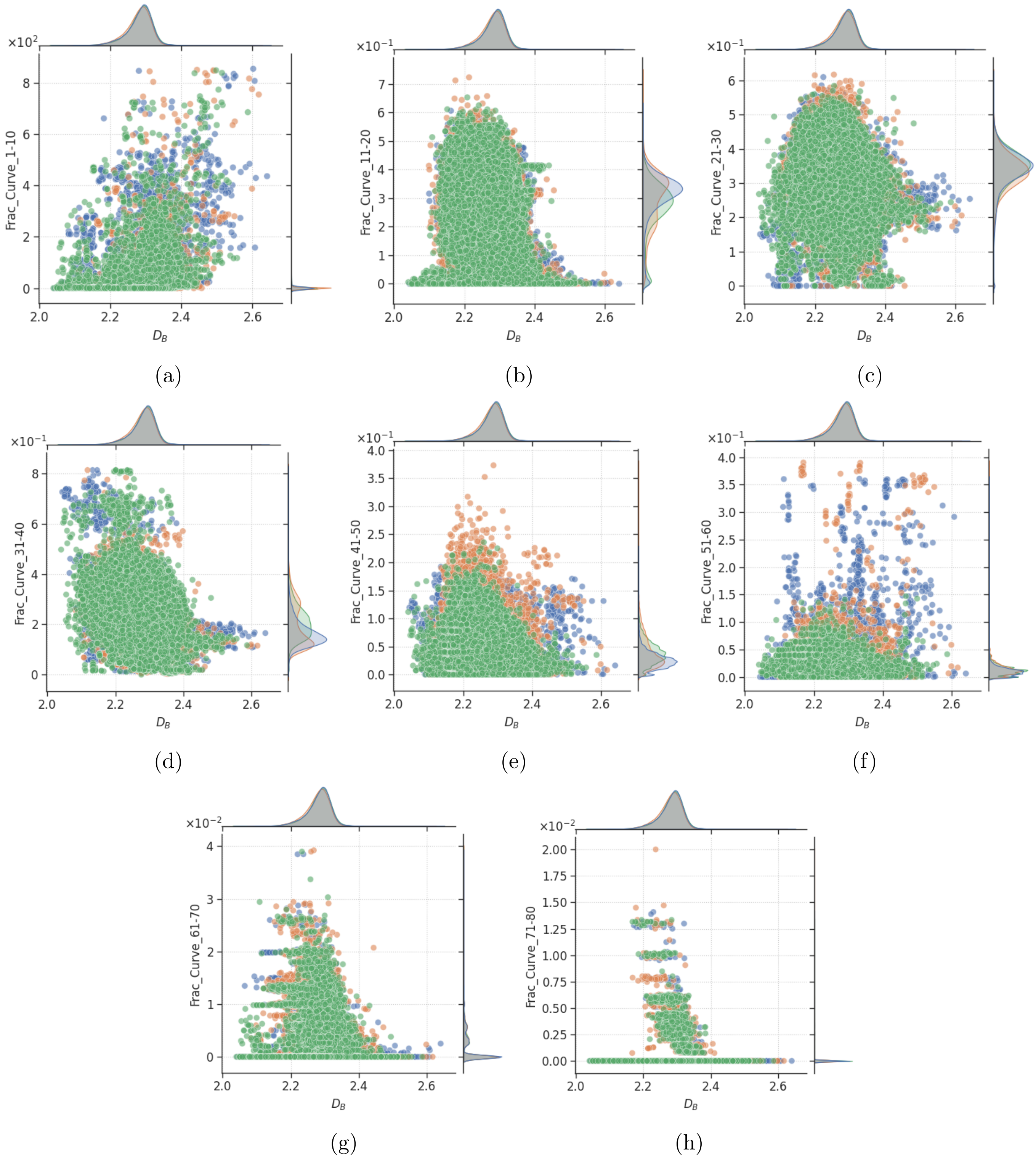

Figure S10: Scatter plots of box-counting dimension and fraction of surface atoms lying on surface with curvatures ranging from (a) 1° to 10°, (b) 11° to 20°, (c) 21° to 30°, (d) 31° to 40°, (e) 41° to 50°, (f) 51° to 60°, (g) 61° to 70°, and (h) 71° to 80° for bimetallic nanoparticles. The blue, orange, and green points correspond to AuPd, AuPt, and PdPt nanoparticles, respectively. The mutual information scores for the relationships are provided in main text Table 1, with higher values indicating greater Scatter plots of dependencies of the features on box-counting dimension.

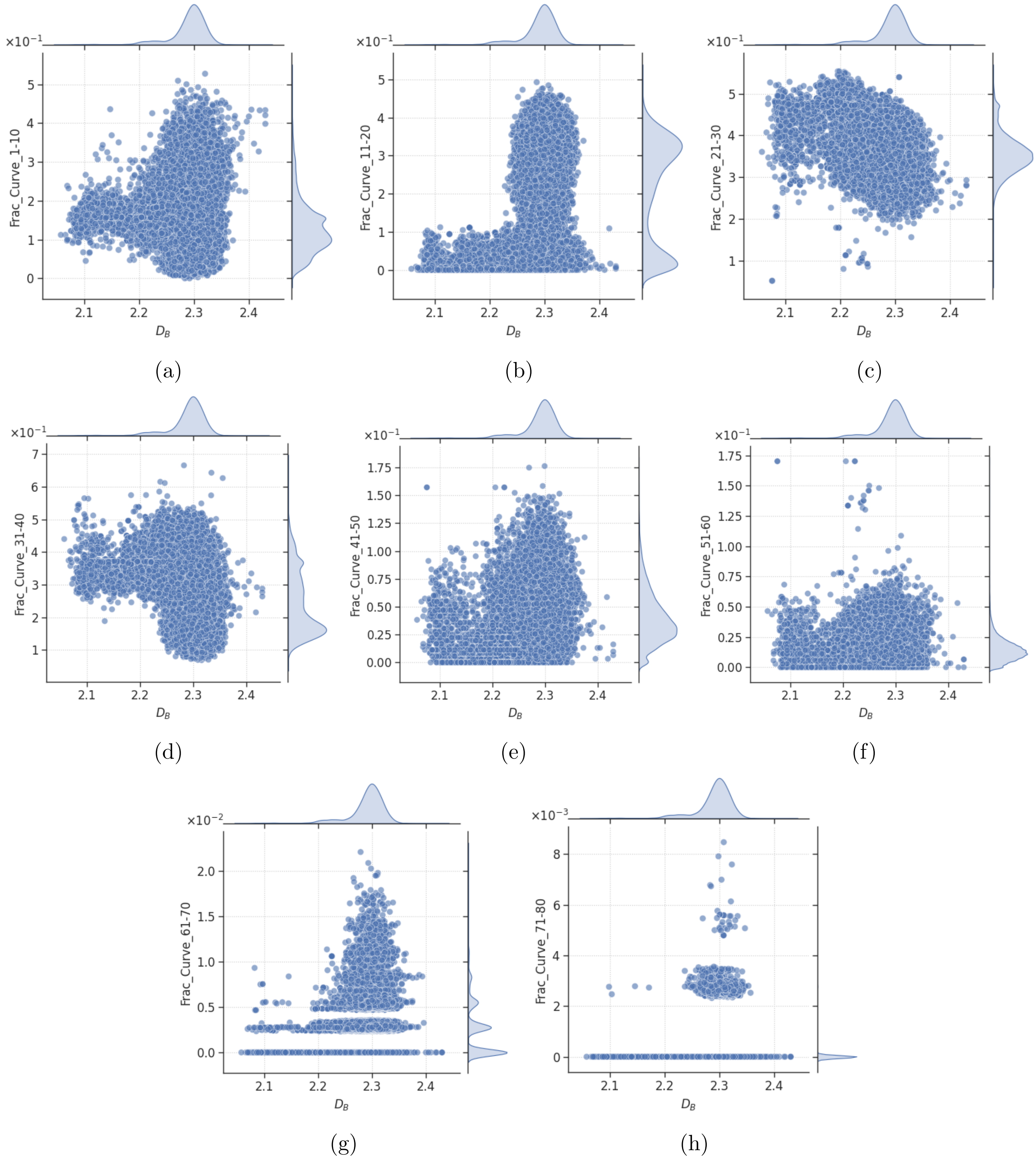

Figure S11: Scatter plots of box-counting dimension and fraction of surface atoms lying on surface with curvatures ranging from (a) 1° to 10°, (b) 11° to 20°, (c) 21° to 30°, (d) 31° to 40°, (e) 41° to 50°, (f) 51° to 60°, (g) 61° to 70°, and (h) 71° to 80° for trimetallic nanoparticles. The mutual information scores for the relationships are provided in main text Table 1, with higher values indicating greater Scatter plots of dependencies of the features on box-counting dimension.

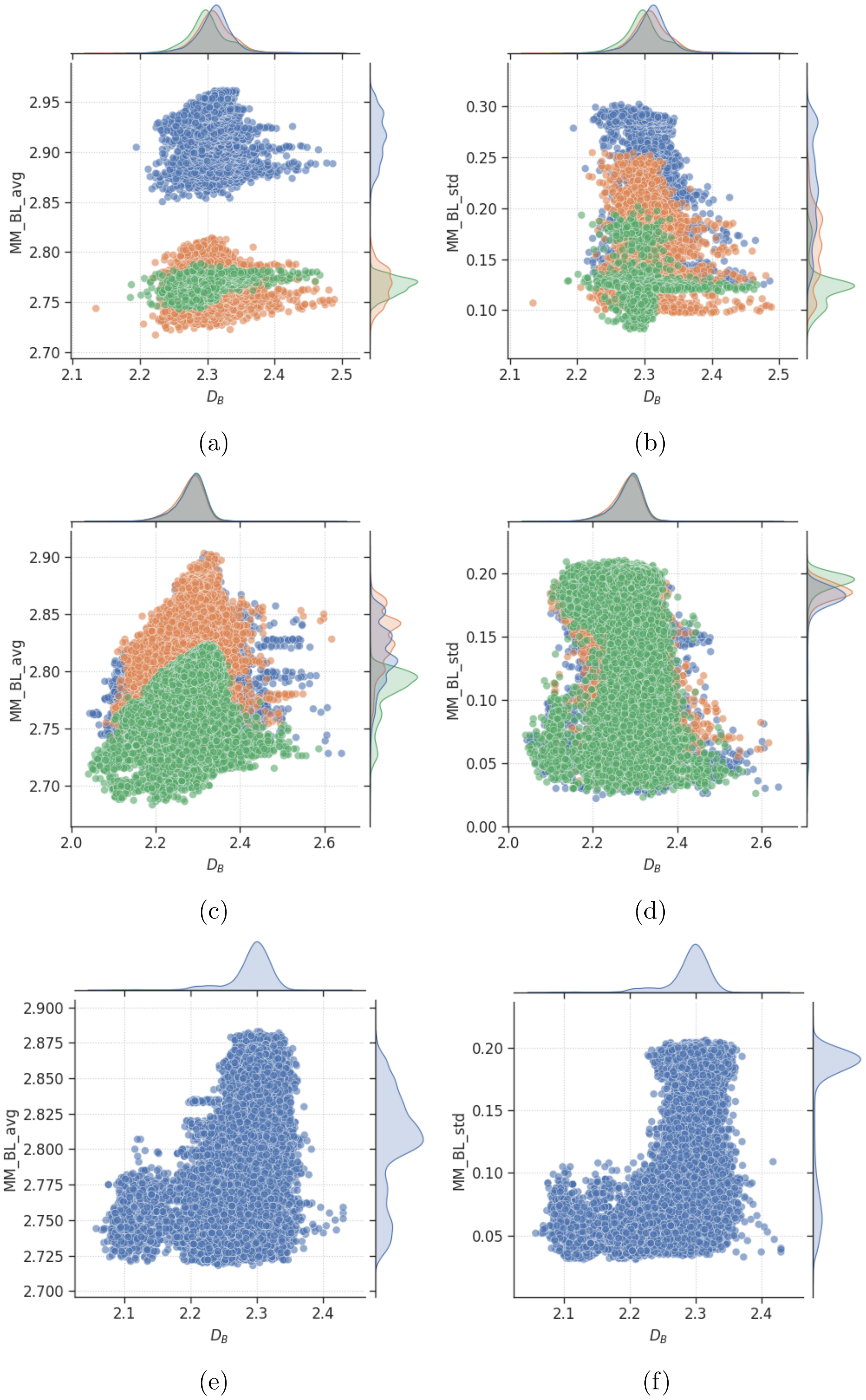

Figure S12: Scatter plots of box-counting dimension and bond length statistics, including average (left) and standard deviation (right) for monometallic (upper, Au = blue, Pd = orange, Pt = green), bimetallic (middle, AuPd = blue, AuPt = orange, PdPt = green), and trimetallic (lower, blue) nanoparticles. The mutual information scores for the relationships are provided in main text Table 1, with higher values indicating greater Scatter plots of dependencies of the features on box-counting dimension.

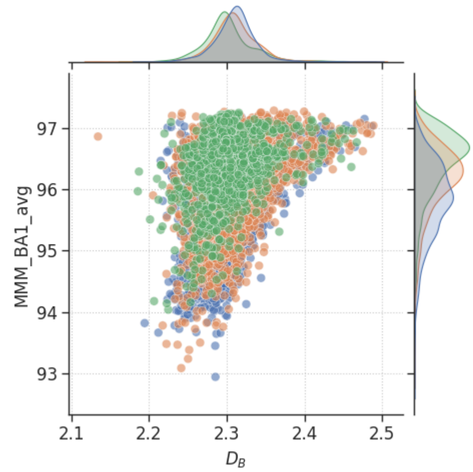

(a)

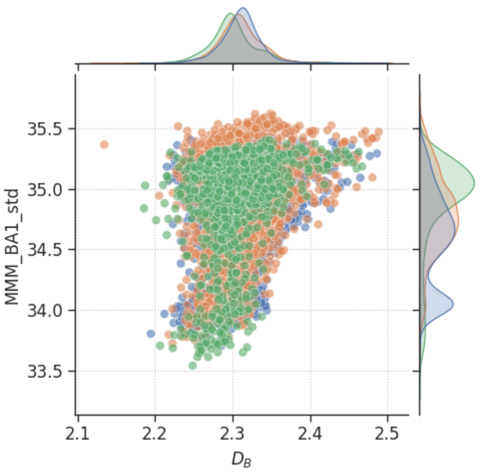

(b)

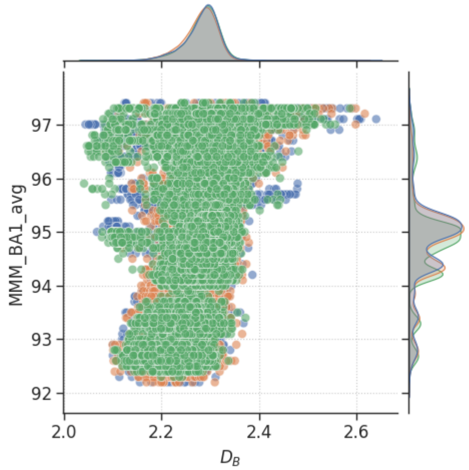

(c)

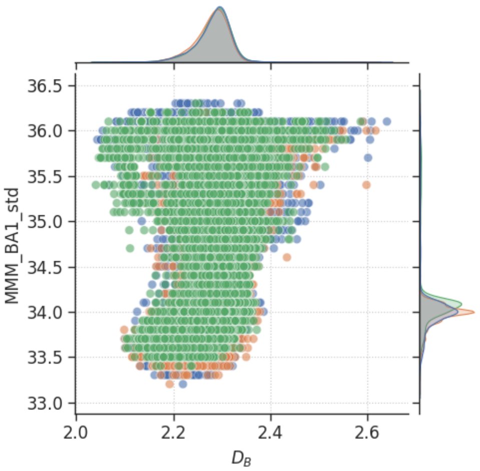

(d)

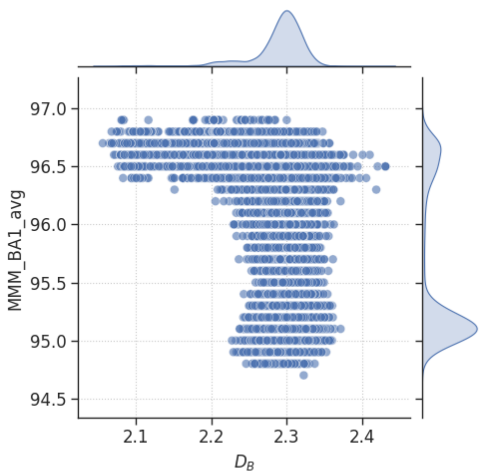

(e)

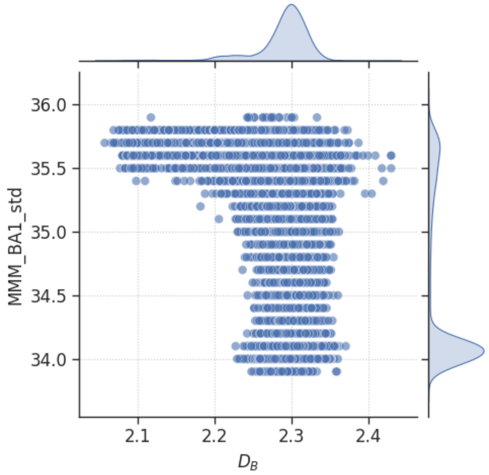

(f)

Figure S13: Scatter plots of box-counting dimension and bond angle statistics, including average (left) and standard deviation (right) for monometallic (upper, Au = blue, Pd = orange, Pt = green), bimetallic (middle, AuPd = blue, AuPt = orange, PdPt = green), and trimetallic (lower, blue) nanoparticles. The mutual information scores for the relationships are provided in main text Table 1, with higher values indicating greater Scatter plots of dependencies of the features on box-counting dimension.

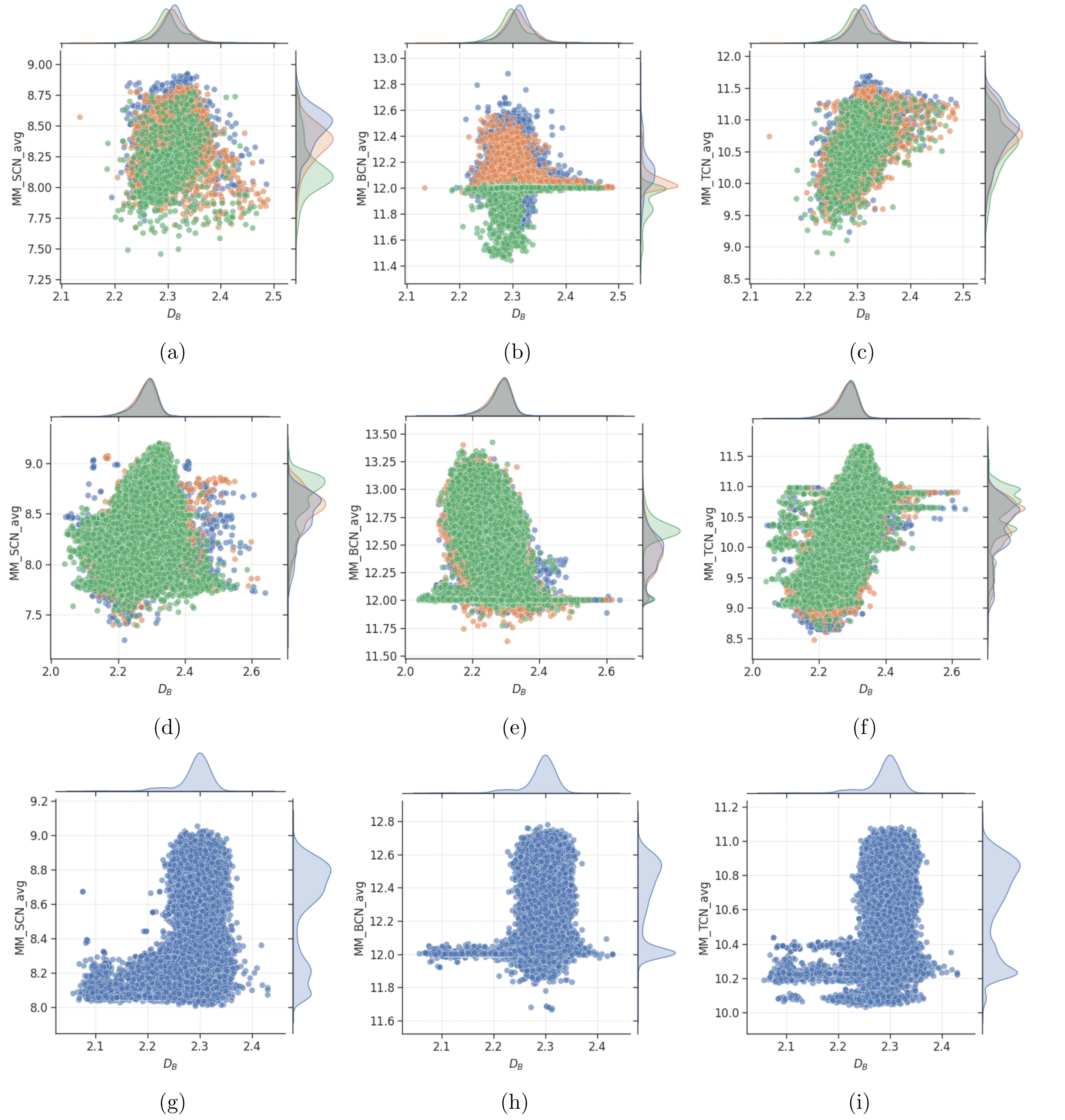

Figure S14: Scatter plots of box-counting dimension and average coordination number of surface (left), bulk (middle) and all (right) atoms for monometallic (upper, Au = blue, Pd = orange, Pt = green), bimetallic (middle, AuPd = blue, AuPt = orange, PdPt = green), and trimetallic (lower, blue) nanoparticles. The mutual information scores for the relationships are provided in main text Table 1, with higher values indicating greater Scatter plots of dependencies of the features on box-counting dimension.

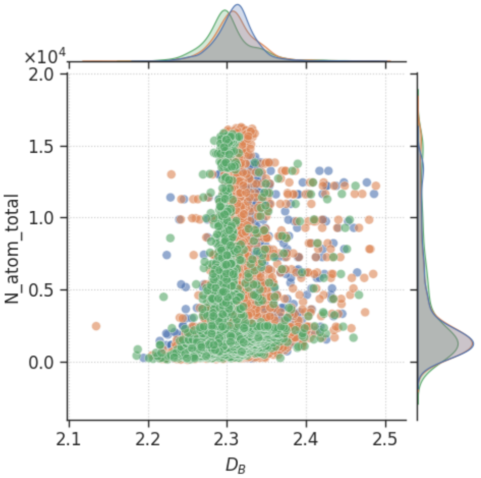

(a)

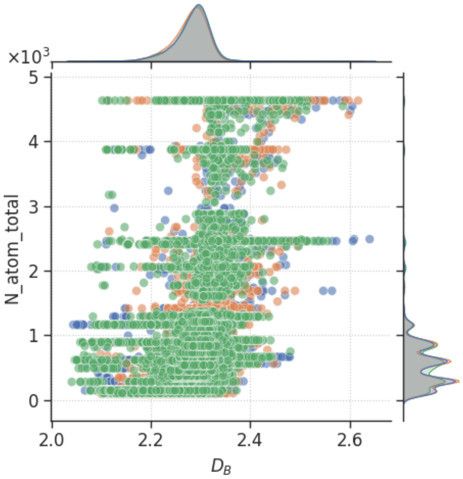

(b)

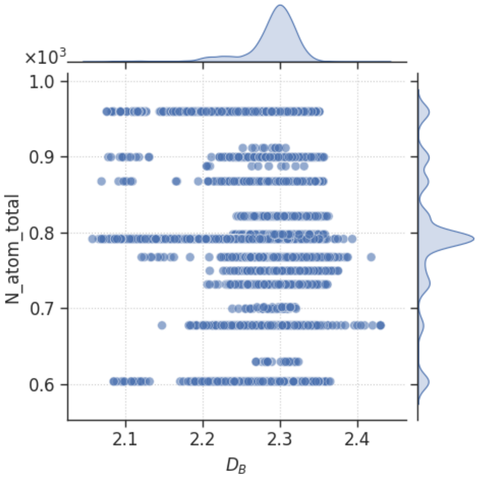

(c)

Figure S15: Scatter plots of box-counting dimension and total number of atoms for (a) monometallic (with Au shown in blue, Pd in orange and Pt in green), (b) bimetallic (with AuPd shown in blue, AuPt in orange and PdPt in green), and (c) trimetallic (blue) nanoparticles. The mutual information scores for the relationships provided in main text Table 1, with higher values indicating greater Scatter plots of dependencies of the features on box-counting dimension.

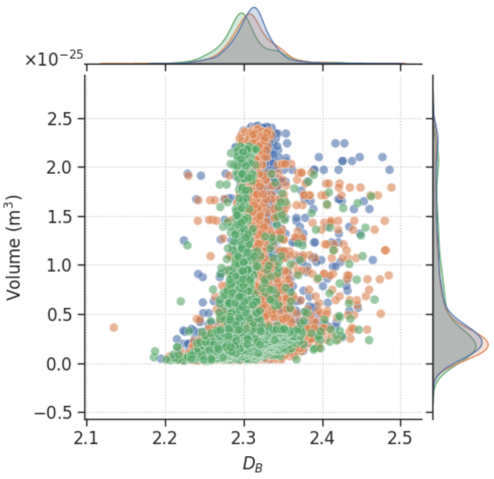

(a)

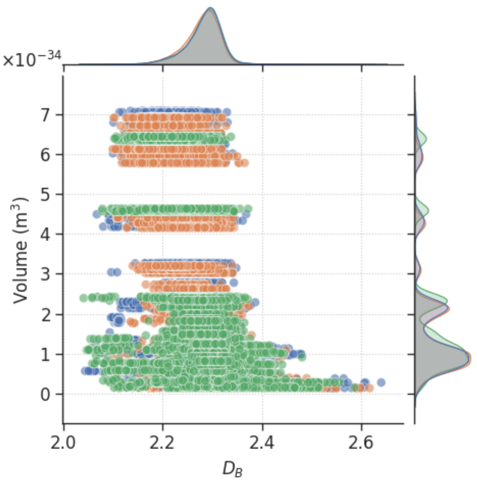

(b)

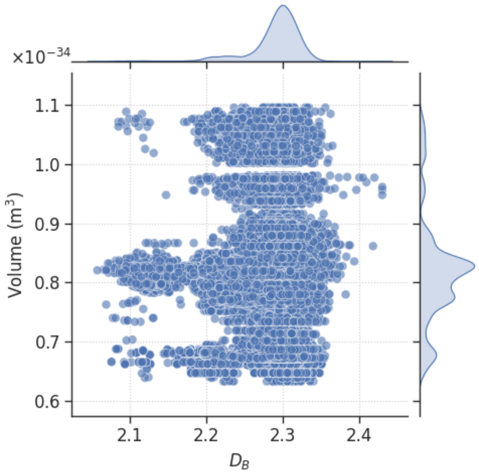

(c)

Figure S16: Scatter plots of box-counting dimension and volume computed based on bulk density assumption for (a) monometallic (with Au shown in blue, Pd in orange and Pt in green), (b) bimetallic (with AuPd shown in blue, AuPt in orange and PdPt in green), and (c) trimetallic (blue) nanoparticles. The mutual information scores for the relationships provided in main text Table 1, with higher values indicating greater Scatter plots of dependencies of the features on box-counting dimension.
